# Development of a multiplex qPCR-based approach for the diagnosis of *Dirofilaria immitis*, *D. repens* and *Acanthocheilonema reconditum*

**DOI:** 10.1186/s13071-020-04185-0

**Published:** 2020-06-22

**Authors:** Younes Laidoudi, Bernard Davoust, Marie Varloud, El Hadji Amadou Niang, Florence Fenollar, Oleg Mediannikov

**Affiliations:** 1 Microbes, Evolution, Phylogeny and Infection (MEPHI), UMR Aix-Marseille University, IRD, APHM, IHU Méditerranée Infection, 19–21, Bd Jean Moulin, 13005 Marseille, France; 2 IHU Méditerranée Infection, 19–21, Bd Jean Moulin, 13005 Marseille, France; 3 Ceva Santé Animale, 10 Avenue de la Ballastière, 33500 Libourne, France; 4 VITROME, UMR Aix-Marseille University, IRD, SSA, APHM, IHU Méditerranée Infection, 19–21, Bd Jean Moulin, 13005 Marseille, France

**Keywords:** Canine filariosis, Multiplex qPCRs, Differential diagnosis, Heartworm disease, *Dirofilaria immitis*

## Abstract

**Background:**

*Dirofilaria immitis*, *D. repens* and *Acanthocheilonema reconditum* are the main causative agents of zoonotic canine filariosis.

**Methods:**

We developed a combined multiplex approach for filaria and *Wolbachia* detection using the *28S*-based pan-filarial and *16S*-based pan-*Wolbachia* qPCRs, respectively, involving a fast typing method of positive samples using triplex qPCR targeting *A. reconditum*, *D. immitis* and *D. repens*, and a duplex qPCR targeting *Wolbachia* of *D. immitis* and *D. repens*. The approach was complemented by a duplex qPCR for the differential diagnosis of heartworms (*D. immitis* and *Angiostrongylus vasorum*) and pan-filarial *cox*1 and pan-*Wolbachia fts*Z PCRs to identify other filarial parasites and their *Wolbachia*, respectively. A total of 168 canine blood and sera samples were used to validate the approach. Spearmanʼs correlation was used to assess the association between filarial species and the strain of *Wolbachia*. Positive samples for both the heartworm antigen-test after heating sera and at least one DNA-positive for *D. immitis* and its *Wolbachia* were considered true positive for heartworm infection. Indeed, the presence of *D. repens* DNA or that of its *Wolbachia* as well as *A. reconditum* DNA indicates true positive infections.

**Results:**

The detection limit for *Wolbachia* and filariae qPCRs ranged from 5 × 10^−1^ to 1.5 × 10^−4^ mf/ml of blood. When tested on clinical samples, 29.2% (49/168) tested positive for filariae or *Wolbachia* DNA. Filarial species and *Wolbachia* genotypes were identified by the combined multiplex approach from all positive samples. Each species of *Dirofilaria* was significantly associated with a specific genotype of *Wolbachia*. Compared to the true positives, the approach showed excellent agreement (*k* = 0.98–1). Unlike *D. immitis* DNA, no *A. vasorum* DNA was detected by the duplex qPCR. The immunochromatographic test for heartworm antigen showed a substantial (*k* = 0.6) and a weak (*k* = 0.15) agreements before and after thermal pre-treatment of sera, respectively.

**Conclusions:**

The proposed approach is a reliable tool for the exploration and diagnosis of occult and non-occult canine filariosis. The current diagnosis of heartworm disease based on antigen detection should always be confirmed by qPCR essays. Sera heat pre-treatment is not effective and strongly discouraged.
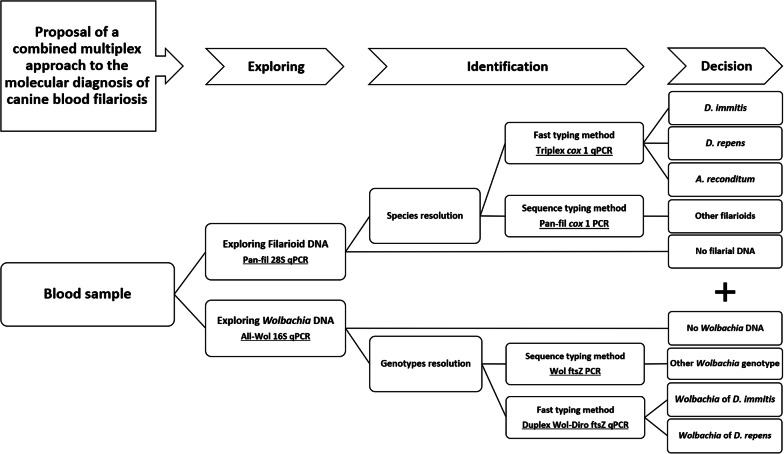

## Background

Canine filariosis includes diseases caused by parasitic nematodes called filariae, belonging to the order Spirurida. There are several species of veterinary and human importance. Dogs seem to be the natural hosts for several species, such as *Dirofilaria immitis*, *D. repens*, *Acanthocheilonema reconditum*, *A. dracunculoides*, *Cercopithifilaria grassii*, *Brugia ceylonensis*, *B. patei*, *B. malayi*, *B. pahangi*, *Onchocerca lupi* and *Thelazia callipaeda* [1–4]. These arthropod-borne filarioids produce blood, cutaneous or mucous microfilariae, where they are available to arthropod vectors [5]. The most common and medically important species affecting dogs are *D. immitis*, *D. repens* and *A. reconditum* [6]. In addition to their veterinary importance, they can also affect human health. *Dirofilaria immitis* causes pulmonary and cardiopulmonary dirofilariosis in humans and dogs, respectively. Cardiopulmonary dirofilariosis usually called heartworm disease has recently been considered as an emerging disease in Europe. In the USA, *D. immitis* is the most important life-threatening parasitic infection in dogs [7]. Elsewhere in the world, particularly in eastern European countries, *D. repens* is the most endemic parasitic nematode causing subcutaneous infection, which is less virulent but more zoonotic than that caused by *D. immitis* [8]*. Acanthocheilonema reconditum* is an occasional zoonotic agent that affects the subcutaneous tissue and the perirenal fat [9, 10] causing a common but clinically less important infection in dogs [11].

Once mature, these filarioids can produce microfilariae circulating in the bloodstream. This larval stage (L1) is also a target for the diagnosis by microscopic detection of the larvae or by detection of their DNA in the host blood [10]. *Dirofilaria immitis*, the agent of heartworm disease, is distributed worldwide and is responsible for heart failure in dogs after colonization of pulmonary arteries and the right ventricle, where it can be fatal if untreated. Due to the gravity of the disease, it remains the most commonly diagnosed filariosis in dogs due to the detection of antigen circulating in the blood [11–13]. Several problems with the current diagnostic methods have been raised, such as morphological confusion between the microfilariae of *D. immitis*, *A. reconditum* and *D. repens.* Commercially available diagnostic kits for the detection of *D. immitis* antigens may also cross-react with filarial and non-filarial nematodes, such as *D. repens*, *A. reconditum* and *Onchocerca* spp. [14], *Spirocerca lupi* and *Angiostrongylus vasorum*, especially the latter which can cause a cross-reaction without prior heat pre-treatment of the sera [15–17]*. Angiostrongylus vasorum*, the agent of French heartworm disease, should also be taken into account in the differential diagnosis of pulmonary disease [17]. This so-called occult heartworm is characterized by the absence of microfilaremia or an amicrofilaremia. This may result from the hostʼs immune response, low parasite load and infertility or, incidentally, the microfilaricidal effect observed in dogs receiving macrocyclic lactone prevention [14]. When the occult heartworm occurs in a co-infection with another filariosis, the diagnosis is even more challenging. The association of the heartworm with *D. repens* infection may result in an unexplained suppressive effect on the production of microfilariae of *D. immitis* [18, 19]. In such cases, the cross-reactivity between *D. immitis* and *D. repens* may result in misdiagnosis. Therefore, there is an urgent need for a more sensitive diagnostic method to detect occult as well as non-occult canine filariosis and to identify the pathogen. Detection of *D. immitis* has gained more and more attention; many trials have been performed for improving the quality of heartworm diagnostic tools, such as the detection of a specific antigen released by these worms [20], or the use of a recombined antigen of *D. immitis* for specific antibody detection [21].

The endosymbiotic intracellular bacteria of the genus *Wolbachia* are associated with some filarial species of two subfamilies of the Onchocercidae: Onchocercinae and Dirofilariinae [22]. These bacteria are host-specific, and each species of filarial worm is associated with a specific bacterial genotype. *Wolbachia* spp. have been targeted for the indirect diagnosis of *D. immitis* infection in dead-end hosts such as humans and cats, whereby the strong reaction of the host against the parasite prevents them to achieving their maturation, and, therefore, the production of microfilariae may not be achieved [23]. In such cases, the detection of filaria-specific *Wolbachia* may indicate a filarial infection and can serve as an alternative diagnostic tool in endemic areas [23, 24]. In around 40–60% of canine heartworm cases, both *Wolbachia* and parasite DNA may be detected using conventional PCR [25–27]. Indeed, the combined detection of *Wolbachia* and *Dirofilaria* DNA was suggested to improve heartworm detection [27].

In the present study, we developed a multiplex real-time PCR-based approach allowing a specific, rapid and simultaneous detection of *D. immitis*, *D. repens* and *A. reconditum* as well as the occult *Dirofilaria* spp. infections in dogs. In addition, it was completed by a duplex real-time PCR-based assay for the simultaneous detection of *D. immitis* and *A. vasorum* as a differential diagnostic for canine heartworms. The approach can be used in routinely in a diagnostic laboratory. We also evaluated the effectiveness of a novel molecular approach to conventional serological diagnosis and assessed the importance of serum heating.

## Methods

### Probes, primers design and PCR amplification protocol

#### Custom protocol and *in silico* validation

First, for each PCR assay, the target gene was chosen to meet the objective of each system. Fasta files were constructed from the sequences of the representative members of the family Onchocercidae or *Wolbachia* genotypes available in the GenBank database. The sequences were aligned using BioEdit v 7.0.5.3 software [28] to reveal the highly conserved inter- and intra-species regions as target regions for primers and probes. This region was submitted to Primer3 online software v. 0.4.0 (http://primer3.ut.ee), in order to determine valuable candidate primers and probes; the selection was based on the criteria for primer and probe design.

Physicochemical characteristics, annealing temperature and the possibility for hairpin, self- and hetero-dimers were tested using free online software Oligo-Analyzer 3.1 [29]. Primer sets and probes were also checked within DNA databases of metazoans (taxid:33208), vertebrates (taxid:7742), bacteria (taxid:2), Canidae (taxid:9608), Felidae (taxid:9682) and humans (taxid:9605) using primer-BLAST [30]. This was completed for all possible forward-reverse and probe-reverse combinations of each PCR system. Primers were synthesized by Eurogentec (Liège, Belgium) and the hydrolysis probe was synthesized by Applied Biosystems^TM^ (Foster City, CA, USA).

#### TaqMan simplex qPCR targeting filarial nematodes

The choice of the large subunit rRNA (*LSU*) gene, also called *28S* gene, was based on several criteria such as: the tandem repetition of about 150 times in the filarial nematode genome, which improves the PCR detectability [31]; availability on GenBank for representatives of all nematode families; and sharing a highly conserved region within the Onchocercidae. The primers qFil-28S-F, qFil-28S-R and a TaqMan® hydrolysis probe (qFil-28S-P) were designed to amplify *28S* gene for most filarial species (Table 1).

**Table 1:** Primers and probes developed in this study

System name	Target gene	Primer and probe name	Sequence (5′-3′)	Assay specificity
Pan-fil *28S* qPCR-based system	*28S* rRNA	qFil-28S-F	TTGTTTGAGATTGCAGCCCA	Filariae
		qFil-28S-P	6FAM-CAAGTACCGTGAGGGAAAGT-TAMRA	
		qFil-28S-R	GTTTCCATCTCAGCGGTTTC	
All-Wol *16S* qPCR-based system	*16S* rRNA	all.Wol.16S.301-F	TGGAACTGAGATACGGTCCAG	*Wolbachia*
		all.Wol.16S.347-P	6FAM-AATATTGGACAATGGGCGAA-TAMRA	
		all.Wol.16S.478-R	GCACGGAGTTAGCCAGGACT	
Triplex TaqMan *cox*1 qPCR-based system	*cox*1	Fil.COI.749-F	CATCCTGAGGTTTATGTTATTATTTT	*D. immitis*, *D. repens* and *A.reconditum*
		D.imm.COI.777-P	6FAM-CGGTGTTTGGGATTGTTAGTG-TAMRA	
		D.rep.COI.871-P	6VIC-TGCTGTTTTAGGTACTTCTGTTTGAG-TAMRA	
		A.rec.COI.866-P	Cy5-TGAATTGCTGTACTGGGAACT-BHQ3	
		Fil.COI.914-R	CWGTATACATATGATGRCCYCA	
Duplex Wol-Diro ftsZ qPCR-based system	*ftsZ*	WDiro.ftsZ.490-F	AAGCCATTTRGCTTYGAAGGTG	Endosymbiotic *Wolbachia* of *D. immitis* and *D. repens*
		WDimm.ftsZ.523-P	6FAM-CGTATTGCAGAGCTCGGATTA-TAMRA	
		WDrep.ftsZ.525-P	6VIC-CATTGCAGAACTGGGACTGG-TAMRA	
		WDiro.ftsZ.600-R	AAACAAGTTTTGRTTTGGAATAACAAT	
Duplex HWs *cox*1 qPCR-based system	*cox*1	Hw.COI.723-F	TCAGCATTTGTTTTGGTTTTT	*D. immitis* and *A. vasorum*
		D.imm.COI.777-P	6FAM-CGGTGTTTGGGATTGTTAGTG-TAMRA	
		A.vas.COI.813-P	6VIC-TGACTGGGAAGAAGGAGGTG-TAMRA	
		Hw.COI.950-R	GCASTAAAATAAGYACGAGWATC	

#### TaqMan triplex qPCR targeting *D. immitis*, *D. repens* and *A. reconditum*

The gene encoding for the cytochrome *c* oxidase subunit 1 gene (*cox*1) was selected for the development of the triplex TaqMan qPCR system targeting *D. immitis*, *D. repens* and *A. reconditum* (Table 1). This choice was based on the availability of *cox*1 for the three species on GenBank. Indeed, the *cox*1 gene is recognized for its high sensitivity (a high copy number relative to the nuclear gene in each cell) [32]. The *cox*1 gene has been described as a “barcode gene” for filarial nematodes [33]. The primers Fil.COI.749 and dg.Fil.COI.914 (Table1) were designed to amplify a 166 bp-long *cox*1 fragment for most members of the Onchocercidae. The system’s specificity was confined to the TaqMan probes, namely P.imm.COI.777 specific to *D. immitis* and P.rep.COI.871 specific to both *D. repens* and “*Candidatus* Dirofilaria (Nochtiella) honkongensis” affecting dogs and humans in Japan [34]. Finally, the probe P.rec.COI.866 is specific to *A. reconditum.* In the triplex TaqMan system, three different dyes were used for specific detection: FAM and VIC with a non-fluorescent quencher-TAMRA confined to *D. immitis* and *D. repens* probes, respectively; Cyanine 5 (Cy5) with a non-fluorescent quencher-BHQ-3 for the *A. reconditum* probe (Table 1).

#### TaqMan duplex qPCR targeting *D. immitis* and *A. vasorum*

The duplex *cox*1-based qPCR was designed (Table 1) with primers Hw.COI.723-F and Hw.COI.950-R to amplify partial *cox*1 gene (227 bp) of both filarial and non-filarial nematodes, including *D. immitis* and *A. vasorum.* The primers were chosen to flank the probe P.imm.COI.777, previously designed for *D. immitis*. In addition, we designed a new probe named A.vas.COI.813-P specific to *A. vasorum.* The TaqMan probes were labelled with FAM and VIC, respectively, with a non-fluorescent quencher TAMRA.

#### TaqMan simplex qPCR targeting *Wolbachia*

The *16S* rDNA gene has been reported as the most commonly used gene for *Wolbachia* phylogeny [35]. The simplex-qPCR was developed and validated *in silico* for the conserved region of the first third of the *16S* rDNA gene. The qPCR system (Table 1) is composed of primers Wol.16S.301f and Wol.16S.478r with the probe Wol.16S.347p targeting all *Wolbachia* lineages.

#### TaqMan duplex qPCR targeting filarial *Wolbachia*

*Wolbachia* ftsZ gene, the homologue of the eukaryotic protein tubulin, provides sufficient discrimination between *Wolbachia* spp. of supergroups C and D found in filarial nematodes, and those of supergroups A and B found in arthropods with a higher divergence between filarial *Wolbachia* of supergroups C and D [36]. The *ftsZ*-based duplex-qPCR was designed with primers WDiro.ftsZ.490f and wDiro.ftsZ.600r targeting filarial *Wolbachia* belonging to supergroup C, which includes those found in *Dirofilaria* sp. However, the specificity of the duplex-qPCR was confined to probes wDimm.ftsZ.523p and wDrep.ftsZ.525p specific to *Wolbachia* sp. of *D. immitis* and that of *D. repens*, respectively (Table 1).

### Run protocols

The simplex, duplex and triplex qPCR reactions were carried out in a final volume of 20 µl, containing 5 µl of DNA template, 10 µl (2×) of Master Mix Roche (Eurogentec, Liège, Belgium). Volume of each primer per reaction was 0.5 µl (50 µM) for the simplex qPCR and 0.75 µl (50 µM) for both the duplex and triplex qPCR, with 0.5 µl of both UDG (1 U/µl) and each probe (20 µM). The final volume was made up to 20 µl using DNAse-RNAse free UltraPure water (Eurogentec, Liège, Belgium). The TaqMan cycling conditions included two hold steps at 50 °C for 2 min followed by 15 min at 95 °C, and 39 cycles of two steps at 95 °C for 30 s and 60 °C for 30 s. These reactions were performed in a CFX96 Touch Real-time PCR Detection System (Bio-Rad, Marnes-la-Coquette, France) after activating the appropriate absorption channels for the dyes used in each qPCR system.

The accumulation of the relative fluorescence units (RFUs) was recorded during the extension step of each qPCR and was used to set-up the cut-off value for each TaqMan system, according to the formula described [37]. The tolerance value was fixed at 5% for all systems. The qPCR reaction was considered positive only if the RFU value was higher than the cut-off value. qPCR data analysis was performed using the CFX Manager Software Version 3 [37].

### Design of conventional PCR primers, amplification and sequencing protocols

In order to complete the molecular identification of filariae and their *Wolbachia* spp., we designed two sets of degenerate primers (Table 2): (i) Fspec.COI.957f and Fspec.COI.1465r targeting a 509-bp fragment of the *cox*1 gene of filarioids; and (ii) Wol.ftsZ.363.f and Wol.ftsZ.958.r targeting a 595-bp fragment of the *ftsZ* gene of *Wolbachia* lineages that may be associated with filarioids. All PCR reactions were carried out in a total volume of 50 µl, consisting of 25 µl (2×) of AmpliTaq Gold master mix, 18 µl of DNAse-RNAse free UltraPure water (Eurogentec, Liège, Belgium), 1 µl of each primer (20 µM) and 5 µl of DNA template (except no-template controls). The thermal cycling conditions were: incubation step at 95 °C for 15 min, followed by 40 cycles at 95 °C for 1 min, 30 s at the annealing temperature (with a different melting temperature for each PCR assay, see Table 2), 72 °C for 45 s for elongation, followed by a final extension step at 72 °C for 5 min (Table 1). PCR amplification was performed in a Peltier PTC-200 thermal cycler (MJ Research Inc., Watertown, MA, USA). Amplicons were purified using the filter plate Millipore NucleoFast 96 PCR kit following the manufacturer’s recommendations (Macherey Nagel, Düren, Germany). Purified DNA was sequenced using the BigDye® terminator v3.3 cycle sequencing kit DNA in line with the manufacturer’s instructions (Applied Biosystems). Sequencing was performed using 3500xL Genetic Analyzer (Applied Biosystems, Foster City, CA, USA) and a capillary electrophoresis fragment analyzer (Applied Biosystems). The nucleotide sequences were assembled and edited using ChromasPro 2.0.0 and were then checked using the Basic Local Alignment Search Tool (BLAST) [38].

**Table 2:** PCR/sequencing primers developed in this study, their characteristics and conditions

System name	Target gene	Primer name	Sequence (5′-3′)	Amplicon size (bp)	Tm (°C)	Specificity
Pan-fil *cox*1 PCR	*cox*1	Fwd.957	ATRGTTTATCAGTCTTTTTTTATTGG	509	52.0	Filariae
		Rwd.1465	GCAATYCAAATAGAAGCAAAAGT			
Wol ftsZ PCR	*ftsZ*	Wol.ftsZ.363.f	GGRATGGGTGGTGGYACTGG	560	59.5	*Wolbachia*
		Wol.ftsZ.958.r	GCATCAACCTCAAAYARAGTCAT			

*Abbreviation*: Tm, melting temperature

### Specificity, sensitivity and system validation

DNA samples summarized in Additional file 1: Table S1 were used for the *in vitro* validation of all PCR systems as follows. Nineteen samples of genomic DNA from filarial parasites were used to validate the pan-filarial *28S* qPCR. DNA from eight strains of *Wolbachia* endosymbionts of *Aedes albopictus*, *Anopheles gambiae*, *Cimex lectularius*, *C. hemipterus* (PL13 strain), *D. immitis* microfilariae, *D. repens*, *Onchocerca lupi*, *Wuchereria bancrofti* and *Brugia* sp. were used for the *Wolbachia 16S-*based qPCR system. *Dirofilaria immitis*, *D. repens* and *A. reconditum* DNA were used to validate the triplex qPCR. *Dirofilaria immitis* and *A. vasorum* DNA were used to validate the duplex qPCR targeting heartworms. Finally, DNA of *Wolbachia* endosymbiont of *D. immitis* and that of *D. repens* were used for the duplex *ftsZ*-based qPCR system.

All PCR systems were tested for their specificity using several nematodes, arthropods, laboratory-maintained colonies as well as human, monkey, donkey, horse, cattle, mouse and dog DNA. DNA samples used to test the sensitivity and specificity of PCR systems are summarized in Additional file 1: Table S1 and Additional file 2: Table S2.

**Fig. 1:**
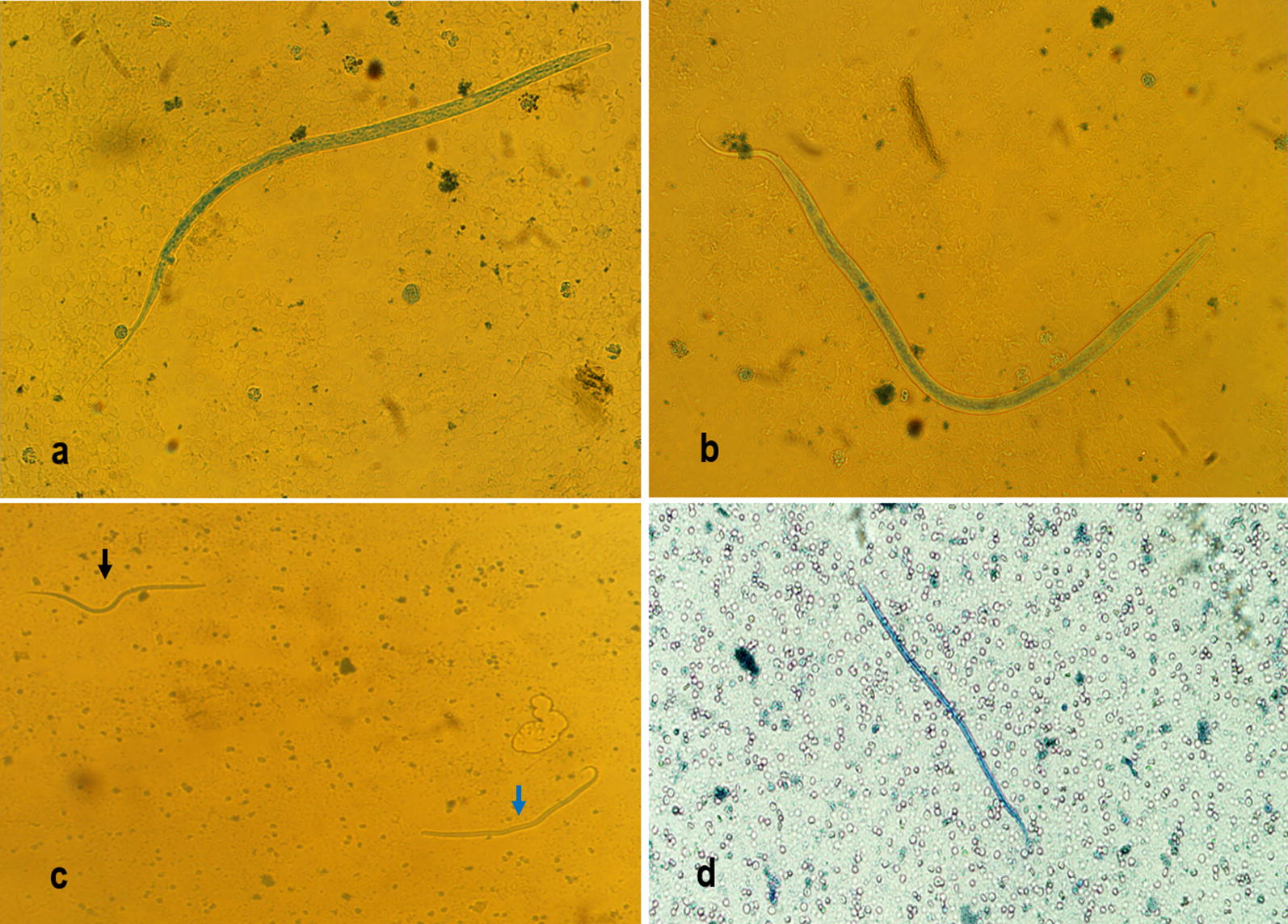
Microfilariae in canine blood by modified Knottʼs test. **a***Dirofilaria immitis* microfilaria. **b***Dirofilaria repens* microfilaria. **c***Dirofilaria immitis* (black arrow) and *D. repens* (blue arrow) co-infection. **d***Acanthocheilonema reconditum* microfilaria

The analytical sensitivity was assessed using a 10-fold dilution of DNA templates, then standard curves and derived parameters (PCR efficiency, slope, Y-intercept and correlation coefficient) were generated using CFX Manager Software Version 3 [37]. The triplex and pan-filarial qPCR systems were challenged in detecting the related numbers of microfilariae of *D. immitis*, *D. repens* and *A. reconditum*. The DNA of each species was obtained from naturally infected canine blood, *D. immitis* (Corsica, 2018), *D. repens* (France, 2018) and *A. reconditum* (Côte d’Ivoire, 2018). First, 1 ml of each blood sample was examined by the modified Knottʼs test [9] to identify the microfilariae species and their number (Fig. 1). Then, the microfilariae concentration was adjusted to 1500 mf/ml by adding Hank’s balanced salt solution (HBSS; Gibco-BRL, Life Technologies, Eragny, France). Thereafter, two extractions were performed from 200 µl: (i) from each separately calibrated sample; and (ii) after mixing an equal volume of each of them to generate a concentration of 500 mf/ml per species. These were used to evaluate the pan-filarial and triplex qPCRs, respectively. Finally, a serial 10-fold dilution of DNA extracted from microfilaremic blood (Corsica, 2018) containing 4033 microfilariae of *D. immitis* was used to assess the analytical sensitivity of both the triplex and duplex (Wol-Diro *ftsZ*) qPCRs in the direct and the indirect detection of single infection with *D. immitis.*

### PCR tools validation by sample screening and identification of filarial infection on biological samples

A pre-existing collection of canine blood and serum samples was used in this study. This included: (i) 8 samples composed of nematode-free laboratory Beagles from the biobank of the Veterinary Research Center of the IHU Méditerranée Infection were used as a negative control group; and (ii) 136 dogs enrolled in March 2017 from Corsica where heartworms are endemic; 7 military working dogs from France recruited on October 2018; and 17 dogs enrolled in April 2018 from Côte d’Ivoire in which blood microfilariae were recorded. Canine blood samples were collected by a veterinarian using cephalic venipuncture into a citrate and serum separator tube. The serum collected and citrate blood were then stored at − 20 °C. These 168 samples were subsequently processed for molecular and serological analysis. Genomic DNA was extracted from the blood and the microfilaria-containing tissues using the Qiagen DNA tissue extraction kit (Qiagen, Hilden, Germany) following the manufacturer’s recommendations. The extracted DNA was eluted in a total volume of 100 µl and stored at − 30 °C.

First, all DNA samples were screened for both filarial and *Wolbachia* DNA using the pan-filarial and pan-*Wolbachia* qPCRs, respectively. Then, partial *cox*1 and *ftsZ* genes were amplified and sequenced according to the previous protocol from all positive samples for filarioids and *Wolbachia*, respectively. Secondly, the fast typing method based on the direct identification of filarial and *Wolbachia* genotypes used the approach combining the triplex *cox*1 and duplex Wol-Diro *ftsZ* qPCR-based systems. Finally, all samples were screened for heartworms using the duplex *cox*1-based qPCR in order to differentiate between *D. immitis* and *A. vasorum* DNA.

The serological analysis was performed on all sera using the DiroCHEK® heartworm antigen test kit (Zoetis, Lyon, France). The test consisting of an enzyme-linked immunosorbent assay sandwich ELISA, targeting the antigen secreted by adult female heartworms [14]. Each serum sample was tested using two different protocols: (i) 200 µl of serum was heated at 104 °C for 10 min followed by centrifugation at 16,000×*g*; (ii) the second protocol was performed without heat-treatment of the sera following the recommendations by Beall et al. [39] regarding the immune complex dissociation to detect any heartworm antigen if present.

In order to evaluate the performance of molecular and serological assays in the absence of the gold standard test (necropsy), we developed the following approach to determine true positive samples. The sample was considered a true positive for heartworm if it was positive for: (i) at least one of the molecular markers of heartworm (DNA of *D. immitis* or its *Wolbachia*); and (ii) a positive antigen test after immune complex dissociation by heating sera. This approach eliminates false-positive serological results that may be obtained by increasing the detection threshold (sensitivity) after heat-treatment of the sera before use, which is subsequently confirmed by the molecular markers specific to *D. immitis*. Once the DNA of *A. reconditum* was identified, the sample was considered a true positive. Finally, samples positive for at least one DNA marker of *D. repens* or DNA of its *Wolbachia* were considered to be true positives.

### Statistical analysis

Results generated through laboratory analysis were recorded in Microsoft Excel (Microsoft Corp., Redmont, USA). In order to assess how *Wolbachia* sp. strains correlated with filarial species, Spearmanʼs correlation coefficient was calculated. In order to evaluate the relevance of each diagnostic approach, the prevalence, correct classification, misclassification, sensitivity, specificity, false positive rate, false negative rate, positive and negative predictive value and Cohen’s Kappa (*k*) measure agreement was calculated. According to the scale of Landis & Koch [40], the agreement quality of Kappa values was interpreted as follows: < 0, no agreement; 0–0.2, slight agreement; 0.2–0.4, fair agreement, 0.4–0.6, moderate agreement; 0.6–0.8, substantial agreement; 0.8–1, almost perfect agreement. Statistical analyses were performed using Addinsoft 2018 (XLSTAT 2018: Data Analysis and Statistical Solution for Microsoft Excel, Paris, France).

## Results

### Validation of the PCR systems

The *in silico* validation revealed that the pan-filarial systems (*28S* qPCR and *cox*1 PCR) were specific for filarial parasites belonging to the subfamilies Dirofilariinae, Onchocercinae, Setariinae, Oswaldofilariinae, Icosiellinae and Waltonellinae. The *16S* qPCR targeting *Wolbachia* strains was specific for all the lineages known so far. However, the *ftsZ* PCR showed specificity for *Wolbachia* strains belonging to supergroups C, D, F and J, that may be associated with filarioids. Likewise, the multiplex qPCRs were also specific for the target species without failure. For each qPCR system, primer melting temperatures were closely identical and were lower than that of the probe. Indeed, the absence of primer-dimer formation and hairpin structures was also confirmed. Furthermore, the specificity was confirmed again by an *in vitro* validation, as shown in Additional file 1: Table S1, where positive reaction was obtained only from the target DNA and no negative control was amplified. Despite using single-species or pooled DNAs, the specific fluorescence signals generated through the multiplex qPCR systems were successfully related to the target DNA (Additional file 3: Table S3, Additional file 4: Figure S1).

### Determining assay performance characteristics

The assay characteristics were assessed for the pan-filarial, the triplex and the duplex qPCR targeting *Wolbachia* sp. endosymbiont of *Dirofilaria* spp. The analytical sensitivity of the pan-filarial qPCR was confirmed three times using *D. immitis*, *D. repens* and *A. reconditum* DNA sharing the same microfilariae concentration. This assay was able to detect up to 1.5 × 10^−4^ microfilariae per ml (mf/ml) (corresponding to 0.75 × 10^−6^ mf/5 μl). Efficiency ranged from 99.1 to 100.7%, with a slope from − 3.34 to − 3.30, Y-intercept values from 21.71 to 21.72 and an *R*^2^ from 0.996 to 0.999 for microfilariae of all species tested (Additional file 5: Table S4, Additional file 6: Figure S2). However, the analytical sensitivity of the triplex qPCR, using pooled DNA of three species, was confirmed by the detection of up to 5 × 10^−1^ mf/ml (corresponding to 2.5 × 10^−3^ mf/5 μl) of each species simultaneously (Additional file 7: Table S5, Additional file 8: Figure S3). qPCR efficiency ranged from 100.4 to 103.7%, with a slope from − 3.30 to − 3.24, Y-intercept values from 32.89 to 33.19 and an *R*^2^ from 0.993 to 0.999. Finally, the analytical sensitivity was confirmed for both the *cox*1-triplex and the *ftsZ* duplex qPCRs in detecting the infection by *D. immitis* (Additional file 9: Table S6).The detection limit was up to 4.03 × 10^−2^ and 4.03 × 10^−1^ mf/ml, respectively (corresponding to 2.01 × 10^−3^ and 2.01 × 10^−2^, respectively) mf/5 μl, qPCR efficiency was 104.8 and 100.5%, respectively, slopes were − 3.212 and − 3.309, respectively. Y-intercept values were 31.17 and 35.98, respectively, and *R*^2^ was above 0.995 for both systems (Additional file 9: Table S6, Additional file 10: Figure S4).

### Molecular diagnostic approaches

**Fig. 2:**
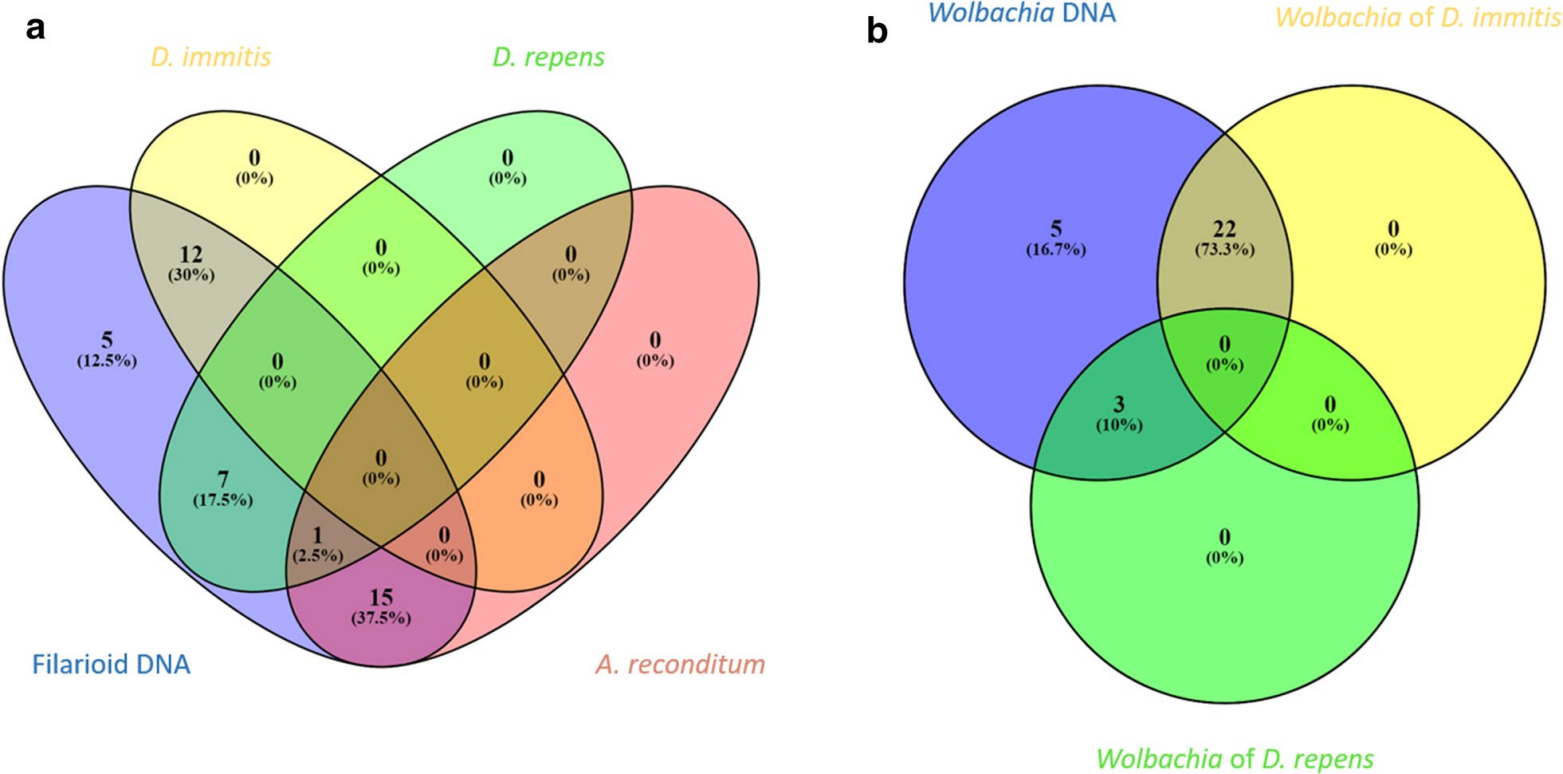
Venn diagram depicting the distribution of positive samples for filariae and *Wolbachia* DNA. **a** Sequence typing method based on the *cox*1 gene of filariae. **b***Wolbachia* genotyping based on the *ftsZ* gene

**Fig. 3:**
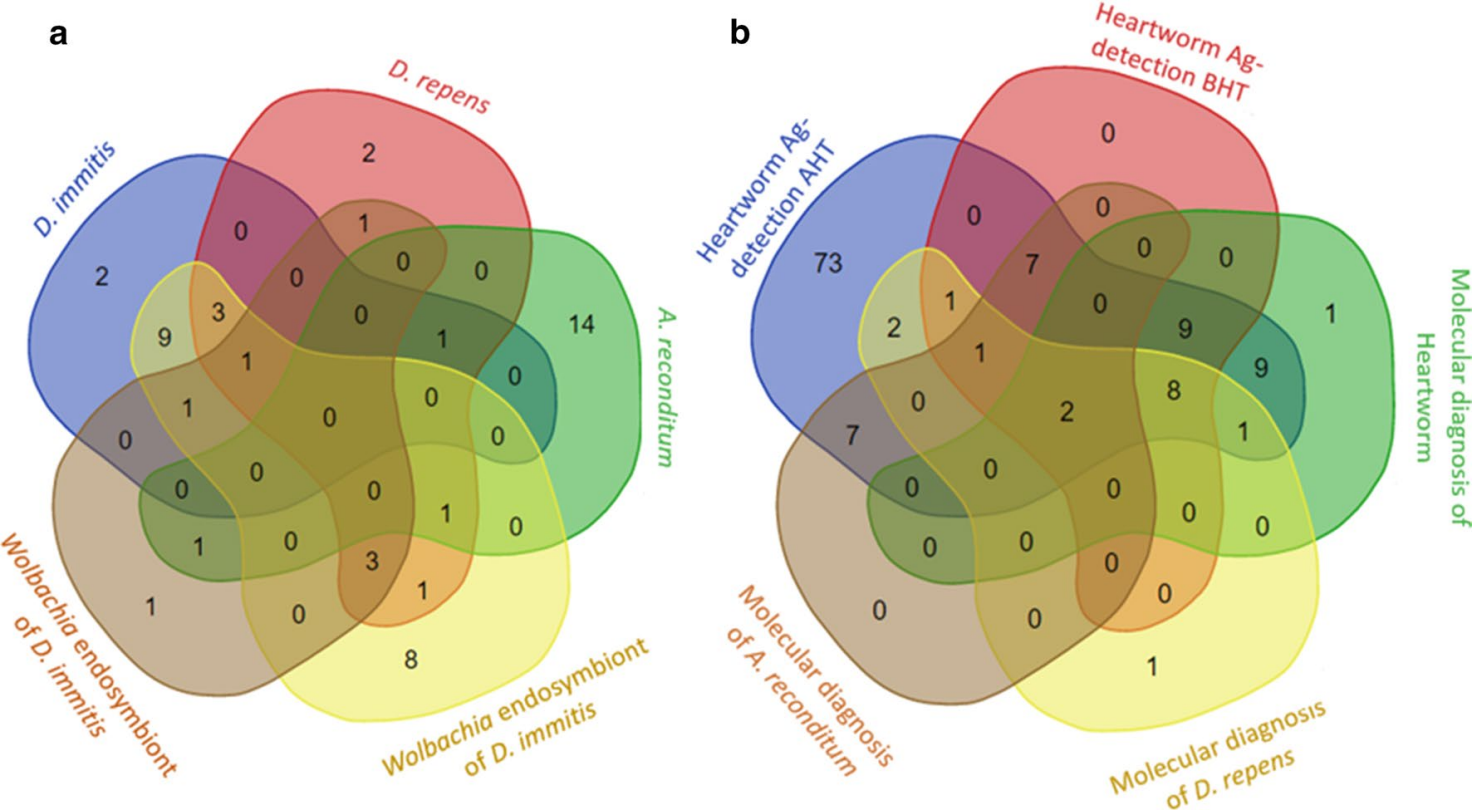
Venn diagram depicting the distribution of positive samples detected by molecular and serological assays. **a** Molecular identification of filariae and associated *Wolbachia* using the multiplex approach. **b** Comparison between molecular diagnosis and heartworm antigen detection before and after heat pre-treatment of sera

Results of molecular screening followed by the sequence typing approach are detailed in Fig. 2a, b and Additional file 11: Table S7. Of the 168 samples tested, 49 (29.17%) were positive for DNA of at least one filaria species or its *Wolbachia* genotype. All positive results were grouped in: (i) 19 blood samples positive only for filarioid DNA; (ii) 9 samples positive only for *Wolbachia* DNA; and (iii) 21 samples positive for both filarial and *Wolbachia* DNA. Although partial *cox*1 and *ftsZ* amplicons were amplified from all positive samples for filariae and *Wolbachia*, *cox*1 gene sequence-based identification allowed the identification of the causative agent of filariosis in 35 (87.5%) out of 40 samples amplified by PCR (Fig. 2a). We here report 12 (30%) cases of *D. immitis*, 7 (17.5%) cases of *D. repens*, 15 (37.5%) cases of *A. reconditum* and one case of both *D. repens* and *A. reconditum* DNA. Noteworthy, the amplicon sequences were obtained separately from this latter case after serial dilution of blood before DNA extraction. However, the *ftsZ* gene sequence-based identification allowed the identification of *Wolbachia* sp. genotype in 25 (83.33%) out of 30 samples amplified by PCR (Fig. 2b). Twenty-two (73.33%) of the *Wolbachia* sequences were closely related to the strain identified in *D. immitis* (GenBank: AJ010272, 99.58%) and 3 (10%) were similar to the strain identified in *D. repens* (GenBank: AJ010273, 99.80%). However, sequence-based identification failed to yield sequences from amplified DNA in 5 cases for each system, which corresponds to 12.5% and 16.7% of filaria and *Wolbachia* DNA samples, respectively. The combined multiplex approach based on the triplex *cox*1 qPCR targeting filariae and the duplex qPCR targeting *Wolbachia*, allowed the detection of 49 samples previously considered positive based on filariae markers (Fig. 3a and Additional file 12: Table S8). The triplex *cox*1 qPCR identified the corresponding species from all the positive samples for filariae (*n* = 40, 100%). Of these, 34 (34.85%) samples had DNA from a single filarial species. *Dirofilaria immitis* was identified in 12 (30%), *D. repens* in 7 (17.5%) and *A. reconditum* in 15 (37.5%) of these samples. Five samples (12.5%) were positive for DNA of two filarial species, of which 4 were positive for *D. immitis* and *D. repens* and one was positive for *D. repens* and *A. reconditum*. Positivity for three filarial species was detected for only one sample (2.5%). The duplex Wol-Diro-*ftsZ* qPCR allowed the identification of *Wolbachia* genotype from all samples positive for *Wolbachia* DNA. Twenty-one samples (21.70%) were positive for *Wolbachia* sp. endosymbiont of *D. immitis*, three samples were positive for *Wolbachia* sp. endosymbiont of *D. repens* and five samples (16.67%) were positive for both strains. Among the 168 samples screened by duplex qPCR for heartworms (*D. immitis* and *A. vasorum*), 17 (10.12%) were positive for *D. immitis* DNA and no *A. vasorum* DNA was detected.

### Link between *Wolbachia* genotype and filarial species within the infected host

The results of the distribution of filarioid markers obtained by multiplex qPCRs are shown in Fig. 3a and Additional file 12: Table S8. Interestingly, most samples which were positive for filarioid DNA were also positive for *Wolbachia* (21/40, 52.5%). Indeed, *Wolbachia* DNA was associated with at least one *Dirofilaria* spp. in 80% (20/25) of the samples having dirofilarial DNA. Analysis of the correlation between *Wolbachia* strains and *Dirofilaria* species are shown in Table 3. Seventy-five percent (9/12) of the samples positive for *D. immitis* DNA alone were also positive for *Wolbachia* genotype known to be associated with this filarioid, which corresponds to a significant correlation (*r* = 0.509, *P* < 0.0001). In addition, there was a significant correlation (*r* = 0.181, *P* < 0.019) between the presence of *D. repens* DNA alone and that of *Wolbachia* strain commonly associated with this filarioid. In general, the presence of *D.* *repens* DNA was correlated with the presence of DNA of both *Wolbachia* strains (*r* = 0.454, *P* < 0.0001). The presence of both *D. immitis* and *D. repens* DNA was associated with the presence *Wolbachia* endosymbiont of *D. immitis* (*r* = 0.244, *P* < 0.002) and also with the presence of both *Wolbachia* strains together (*r* = 0.175, *P* = 0.023). On the other hand, 29.63% (8/27) of the samples harboring the *Wolbachia* endosymbiont of *D. immitis* were free of filarioid DNA (Fig. 3a) and 25% (2/8) of the samples positive for *Wolbachia* endosymbiont of *D. repens* DNA were also free for filarioid DNA. No correlation was observed between *A. reconditum* and *Wolbachia* strains.

**Table 3:** Spearman correlation matrix depicting the strength of association between filaria and *Wolbachia* species within infected dogs

Groups of *Wolbachia* DNA	Filarial DNA	*D. immitis* and *D. repens* DNA	Single-species DNA of *D. immitis*	Single-species DNA of *D. repens*	*A. reconditum* DNA
*Wolbachia* DNA	0.506 (< 0.0001)	0.284 (0.0002)	0.565 (< 0.0001)	0.447 (< 0.0001)	− 0.053 (0.490)
Both *Wolbachia* of *D. immitis* and *D. repens* DNA	0.313 (< 0.0001)	0.175 (0.023)	0.087 (0. 259)	0.454 (< 0.0001)	− 0.059 (0.449)
Single-species DNA of *Wolbachia* ex *D. immitis*	0.478 (< 0.0001)	0.244 (< 0.002)	0.509 (< 0.0001)	0.079 (0.381)	− 0.072 (0.335)
Single-species DNA of *Wolbachia* ex *D. repens*	0.334 (< 0.0001)	− 0.024 (0.614)	− 0.037 (0.630)	0.181 (0.019)	0.104 (0.180)

*Note*: The first number represents the correlation coefficient. Values close to zero reflect the absence of correlation. The associated *P*-values are in parentheses

### Heartworm antigen detection and infection status

Of the 168 dog sera tested for heartworm antigen, 16.67% (28) were positive before pre-treatment of the sera and were grouped into three groups (Fig. 3b): (i) 9 (5.36%) were mono-infected by heartworm and were positive for both *D. immitis* and its *Wolbachia* sp. DNA except one, which was positive for *D. immitis* DNA only; (ii) 10 (5.95%) were samples co-infected with at least one other filarioid detected by PCR, comprising 8 (4.76%) positive for *D. repens* and *D. immitis* and 2 (1.19%) positive for *D. immitis*, *D. repens* and *A. reconditum*; and (iii) 9 (5.39%) were positive for filariae other than *D. immitis*, with 7 (4.17%) samples positive for *A. reconditum* DNA only, 1 (0.6%) positive for DNA of *Wolbachia* endosymbiont of *D. repens* and 1 (0.6%) positive for DNA of both *A. reconditum* and *Wolbachia* endosymbiont of *D. repens*.

Once the heat pre-treatment of sera was performed, the rate of positive samples increased up to 71.43% (*n* = 120). Of these, 39.17% (*n* = 47) harbored at least one DNA marker of filarial parasites or their *Wolbachia*. However, two samples (1.19%), one positive for *Wolbachia* endosymbiont of *D. immitis* by qPCR and the other positive for *D. repens* by qPCR, remained serologically negative. For 73 samples (43.46%) no filarioid marker was detected; these were considered positive for unknown antigens (Fig. 3b and Additional file 13: Table S9). No positive results in the negative control group for both serological and molecular assays were obtained.

### Performance characteristics comparison of the diagnostic tools

Once the true positive samples for each filariosis were determined, the diagnostic value was evaluated for each test in the specific detection of filariosis. The sequence typing approach combining the identification of the filariae and *Wolbachia* allowed the diagnosis of heartworm infection in 86.21% (25/29) of cases, which corresponds to a specificity of 82.8% and a sensitivity of 99.3%, thus resulting in an almost perfect agreement with the true positive rate (*k* = 0.87). Compared to the gold standard, the approach combining the multiplex qPCR systems detected one more positive sample for *Wolbachia* endosymbiont of *D. immitis* (Fig. 3b). This approach showed a sensitivity of 100% and a specificity of 99.3%, with an almost perfect agreement (*k* = 0.98) (Additional file 14: Table S10), whereas the detection of heartworm antigen prior to heat pre-treatment of sera showed a sensitivity of 65.5% and a specificity of 93.3%, corresponding to moderate agreement (*k* = 0.6). Additionally, the heat pre-treatment of sera allowed the detection of 71.4% (*n* = 120) including 24.17% (*n* = 29) positive for *D. immitis* infection, 15% (*n=* 18) positive for filariae other than *D. immitis* and 60.83% (*n=* 73) without molecular markers of filariae. The performance characteristics of this tool in detecting heartworm infection when the sera were heated were 100% sensitivity and 34.5% specificity with a slight agreement (*k* = 0.15) (Additional file 14: Table S10). Taking the combined multiplex approach as the gold standard, the sequence typing method had a specificity of 100% and a sensitivity of 62.6% and 94.1% for the detection of *D. repens* and *A. reconditum*, respectively. A substantial (*k* = 0.75) and an almost perfect (*k* = 0.97) agreement with the gold standard test was observed for the detection of *D. repens* and *A. reconditum*, respectively (Additional file 15: Table S11).

## Discussion

### qPCR system validation and assay performance characteristics

The newly developed PCR assay systems have shown specific detection of the target DNA for which they were designed. The pan-filarial *28S* qPCR system aims the detection of filarial DNA from biological samples. It has been adapted for the detection of the filarial parasites known to date, such as members of the subfamilies Dirofilariinae and Onchocercinae parasitizing mammals, reptiles and birds, and those of the subfamily Setariinae, confined to large mammals, and Oswaldofilariinae parasites of reptiles, and amphibian parasites of the subfamilies Icosiellinae and Waltonellinae [5]. The *LSU* rRNA (*28S*) gene targeted by this system is known for its conserved regions between the filarial species [41]. The second qPCR system was customized for the detection of *Wolbachia* DNA irrespective of their lineages. It targets the first part of the *16S* gene which is highly conserved between *Wolbachia* lineages [35]. Another qPCR system for *Wolbachia* targeting the *16S* gene has been proposed as a complementary diagnosis from human blood of the lymphatic filariosis caused by *Wuchereria bancrofti* [42].

In addition to being specific, the multiplex qPCRs were discriminatory towards targeted DNA without failure (Additional file 3: Table S3). These features are directly related to the choice of the target genes, which offer sufficient discrimination between species, as is the case with the *cox*1 gene representing a nematode barcode [34, 43] used for the development of the triplex qPCR for *D. immitis*, *D. repens* and *A. reconditum* and the duplex qPCR targeting *D. immitis* and *A. vasorum* agents of heartworm diseases. *Wolbachia ftsZ* gene, mainly used for the characterization of *Wolbachia* supergroups [36], was used for the development of the duplex qPCR for both *Wolbachia* genotypes associated with *D. immitis* and *D. repens*. A real time PCR for *Wolbachia* endosymbiont of *Brugia pahangi* targeting the same gene has been described [44].

It is worth noting that molecular diagnosis combining the detection of filarial and *Wolbachia* DNA is an improvement and a tool for evaluating treatment protocols targeting filariae and *Wolbachia* [44]. In the present study, the analytical sensitivity of the new qPCR assays ranged from 99.3% to 107.6%, with the slope value of the standard curves ranging from − 3.34 to − 3.15 and coefficients of determination (*R*^2^) higher than 0.99. These characteristics are directly derived from the design protocol, where the formation of heterodimers and hairpins inside and between primers and probes was avoided. Primer sets share a similar melting temperature which is lower than that of the probes, offering a better sensitivity of the qPCR reaction.

The sensitivity of the pan-filarial *28S* qPCR system was much higher than the triplex qPCR for the detection of *D. immitis*, *D. repens* and *A. reconditum* DNA, where the detection limit was 1.5 × 10^−4^ and 5 × 10^−2^ mf/ml, respectively. Indeed, the reference baseline for mitochondrial DNA retrieved from the EZ1 DNA-tissue kit was at 41.4 copies per nuclear genome [45]. Estimated genomic rRNA copy number of 150 in *B. malayi* [32] suggests that the *28S* rRNA gene enables a high amplification efficiency rather than the mitochondrial *cox*1 gene of filarial nematodes. However, the sensitivity of the triplex qPCR in detecting single-species DNA of *D.* *immitis* was much higher than that of the duplex *ftsZ* qPCR in detecting single-species DNA of *Wolbachia* endosymbiont of *D. immitis*, where the detection limit was 4.03 × 10^−3^ and 4.03 × 10^−1^ mf/ml, respectively. Rao et al. [42], reported that filarial DNA is more frequently detected than *Wolbachia* DNA from *W. bancrofti* microfilaremic blood using qPCR assays. The difference of sensitivity could be explained by the weaker infection density by *Wolbachia* at this parasite larval stage [42].

### Molecular diagnostic approaches

Here, we developed and assessed two molecular approaches in detecting and identifying canine filariosis. The first combined the screening and sequence typing of both filarial and *Wolbachia* DNA. The genomic DNA was identified with an almost perfect specificity ranging from 99.3 to 100%. However, the sensitivity ranged from moderate (62.5%) to perfect (94.1%) regarding the presence or absence of co-infection. Overlapping peaks corresponding to different nucleotides on electropherograms of the sequenced samples suggest co-infection [46]. The second approach combines two multiplex qPCR systems targeting *A. reconditum*, *D. immitis*, *D. repens* and the *Wolbachia* genotypes associated with the latter two species. All samples were positive for at least one molecular marker which were detected and identified with an almost perfect sensitivity and specificity using this approach. This method is fast, simple to use, sensitive and highly specific in detecting occult and non-occult filariosis within the infected hosts. The present results reinforce the utility of multiplex qPCR in detecting co-infections, confirm the resolution limits of the sequence typing method in the identification of co-infections [47], and avoiding the sequencing procedure needed using PCR with filaria generic primers [48].

### Linkage between *Wolbachia* strains and filarial species within the infected host

As expected, *Wolbachia* DNA was significantly associated with *Dirofilaria* species in 80% (20/25) of the samples positive for at least one *Dirofilaria* spp. DNA, reinforcing the idea that this endosymbiosis relationship is present in *Dirofilaria* spp. and not in *A. reconditum* [27]. Of the samples positive for *D. immitis* DNA, 75% (9/12) were also found to be positive for the *Wolbachia* genotype known to be associated with this filarioid, resulting in a significant correlation (Table 3). As previously reported, *Wolbachia* DNA was detected in 64.0% of the samples positive for *D. immitis* [27], and in 81.6% of the samples positive for *D. repens* [49]. In the present study, we investigated the link between *Wolbachia* genotype and *D. repens* infection. The samples positive for a single-species DNA of *D. repens* had a significant correlation with the *Wolbachia* genotype known to be associated with this filarioid. This result corroborates the data by Vytautas et al. [49]. Interestingly, the presence of the single-species DNA of *D. repens* was also strongly correlated with the presence of both *Wolbachia* strains associated with *Dirofilaria* spp. This association could be explained, either by the presence of an occult co-infection with *D. immitis*, or by an exchange of *Wolbachia* strains between *Dirofilaria* spp. The first suggestion is supported by the fact that co-infection of *D. repens* and *D. immitis* is often associated with an occult form. This phenomenon results from a competitive suppression between microfilariae species [19]. On the other hand, *Wolbachia* sp. of *D. immitis* was detected in 29.63% (8/27) of the samples in which *D. immitis* DNA was not detected and, in the same samples, an antigen was detected after heat pre-treatment of sera. This result confirms the possibility to detect *Wolbachia* DNA in occult infections. The utility of *Wolbachia* as a diagnosis target for the occult heartworm disease has been demonstrated in the dead-end host, such as humans and cats, where the parasite cannot achieve its maturation and the infection might be amicrofilariaemic [23]. However, the second suggestion related to the exchange (horizontal transfer) of *Wolbachia* strains between *Dirofilaria* species is in contrast with the published data. *Wolbachia* transmission principally occurs *via* eggs of female worms (vertical transfer) [36, 50]. The vertical transfer of *Wolbachia* leads to the specialization of the host-symbiotic relationship [51]. Taylor et al. [52] have indicated that experimental crosses between *B. pahangi* and *B. malayi* have demonstrated *Wolbachia* transmission through female worms only [52]. Theoretically, exchange of *Wolbachia* between *D. immitis* and *D. repens* is hardly possible in natural conditions, because these filariae do not share the same site and the adult worms will not have contact inside the host organism [53]. In addition, it has been reported that each genotype of *Wolbachia* has a specific filarial host [36], and that live worms can release their *Wolbachia* endosymbionts into host tissues [52]. We believe that the presence of a specific genotype of *Wolbachia* is a reliable marker for the presence of its filarial host.

### Performance characteristics of the heartworm antigen detection tests

In the present study, all diagnostic approaches did not react with samples from the negative control group. We assessed the diagnostic value of LISA (DiroCHEK®) in detecting heartworms. The direct exploration of heartworm antigen from sera without heating the sera showed a moderate performance, with sensitivity and specificity values of 65.52% and 93.3%, respectively. Positive antigen tests were obtained from 19 out of 29 (65.52%) of the samples determined as true positives for heartworm and these often harbored both *D. immitis* and its *Wolbachia* sp. DNA. However, 10 samples (34.38%) of which 8 (80%) harbored only DNA of the *Wolbachia* endosymbiont of *D. immitis*, remained undetected by serology. The lack of sensitivity of this assay was unexpected. This may be due in part to the presence of juvenile parasites, which do not produce detectable antigens [54]. Nevertheless, similar discordances have recently been reported, where 41 (38.7%) positive PCR-confirmed microfilaremic samples were negative for heartworm antigen [55]. Therefore, filarial *Wolbachia* interact with the host by activating the Th1 type protective-immune response [56], which could be implemented in the clearance of heartworm antigens.

Finally, nine out of 28 (32.14%) samples were positive for *A. reconditum* and/or *D. repens*; these filariae are known to generate a cross-reactivity in heartworm antigen tests even in the absence of heat treatment of the sera [14]. However, 29 (96.67%) of the samples positive for at least one molecular heartworm marker tested positive for heartworm antigen after heating the sera; this step has recently been added to improve the sensitivity of this test under certain conditions [15, 41]. In contrast, the heat pre-treatment of sera strongly altered the specificity of the heartworm antigen test. Cross-reactivity was observed overall in the samples positive for any filarial parasite, as well as in samples positive for unknown antigens. Filarial (*D. repens* and *A. reconditum*) and non-filarial nematodes (*A. vasorum* and *S. lupi*), are known to cross-react with heartworm antigen tests [15, 57]. Therefore, molecular and serological diagnosis of *A. vasorum* from canine blood showed a close efficiency [58]. Regarding the life-cycle of *A. vasorum* [59], the absence of its DNA from all blood samples tested in the present study is not sufficient enough to rule out infection. However, the qPCR targeting *A. vasorum* developed in this study can be used as a simplex for detecting parasite larvae from other biological samples, such as faeces, pharyngeal swabs as well as in the intermediate hosts. In the absence of circulating microfilaria, the antigen detection can neither confirm nor exclude the occult heartworm in area endemic for the other species that cross-react with the test, as is the case of *D. repens* (Fig. 2). *Dirofilaria repens* microfilariae induce a suppressive effect on those of *D. immitis* [18] which induces the occult form of the latter. The European Society of Dirofilariosis and Angiostrongylosis (ESDA) does not recommend routine heat pre-treatment of sera in an area endemic for these parasites; this is recommended for use only to resolve the discrepancy between other tests, especially when a dog is positive for the microfilaria test and negative for serology, or to confirm a suspicion of clinical disease suggestive of microfilaremia [54].

## Conclusions

**Fig. 4:**
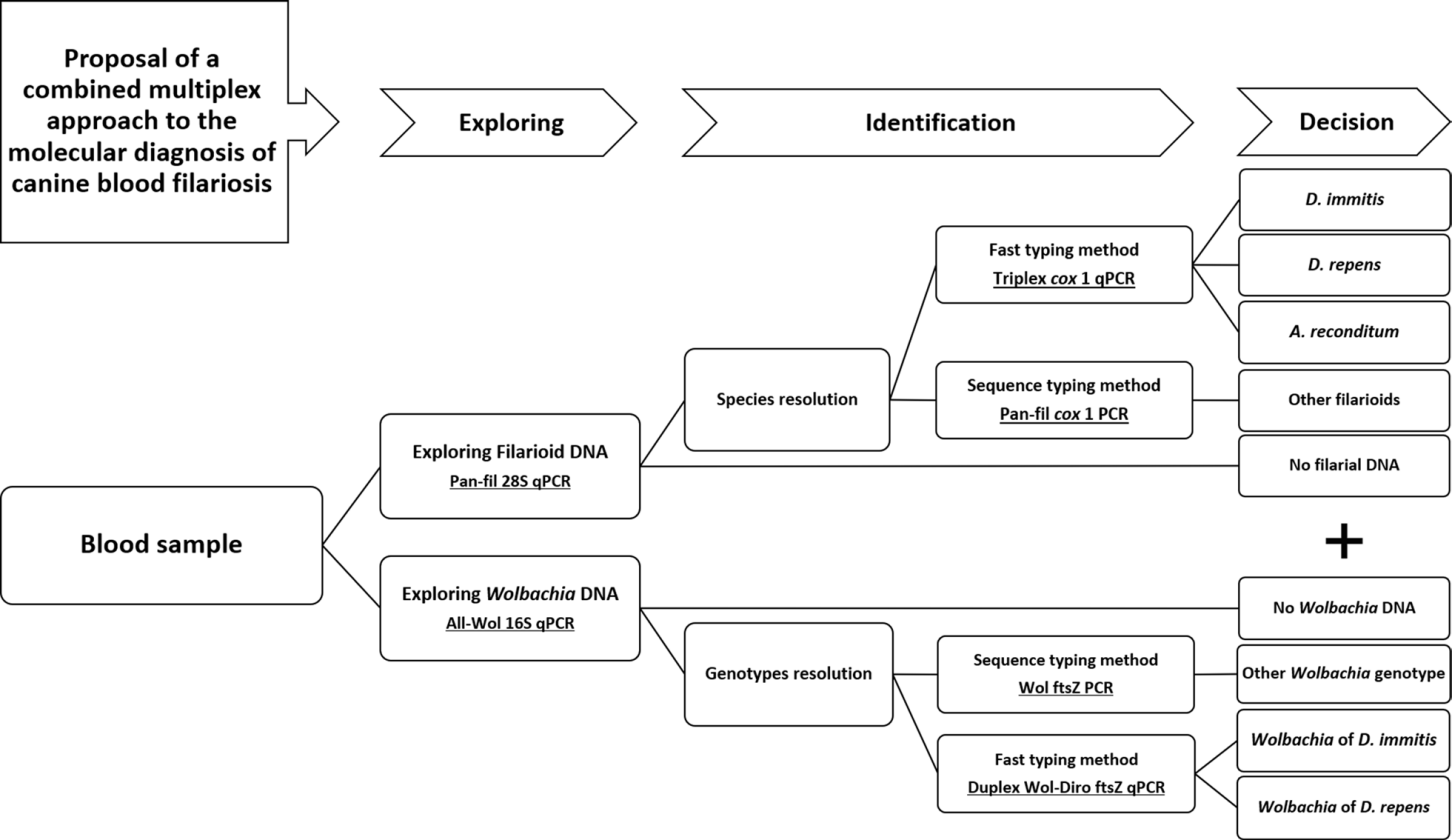
Diagram showing the diagnostic procedure proposed in this study

The molecular approach developed herein represents an improvement in the diagnosis of canine filariosis. It relies principally on TaqMan multiplex qPCR technologies. We encourage researchers to follow the molecular procedure summarized in Fig. 4. The approach allows the detection of filarial parasites as well as their *Wolbachia* endosymbionts at the family level from canine blood. Furthermore, we have implemented a highly sensitive and specific triplex qPCR assay for the simultaneous detection of *D. immitis*, *D. repens* and *A. reconditum*, the most frequent agents of canine filariosis. A duplex qPCR is presented for the simultaneous identification of *Wolbachia* genotypes from *D. immitis* and *D. repens* as a complementary diagnostic of canine dirofilariosis and their occult forms. Two primer sets are proposed for PCR/sequencing of filariae and *Wolbachia* DNAs. Finally, the approach is complemented by a duplex qPCR for *D. immitis* and *A. vasorum*, agents of canine heartworm disease. Moreover, this approach is useful in epidemiological surveillance, in diagnosis and therapeutic follow-up of both filarial parasites and their *Wolbachia* endosymbionts. The specific detection of *Wolbachia* genotypes could be used for the diagnosis of filariosis and the assessment of related pathogeny within dead-end hosts, as is the case of *D. immitis* infection in humans and cats. Heartworm antigen testing without heat treatment of the sera is not reliable in an endemic area with other filarial species, including *A. reconditum* and *D. repens*. We discourage the use of heat pre-treatment of sera, which significantly alters the specificity of the assay due to the cross-reactivity between many filarial and non-filarial nematodes and the possibility of false-positive results that may induce unnecessary heavy treatment of heartworm disease.

## Supplementary information

**Additional file 1: Table S1.** DNA used as a positive control in this study.

**Additional file 2: Table S2.** DNA used as a negative control in this study.

**Additional file 3: Table S3.***In vitro* validation of the triplex *cox*1-based qPCR.

**Additional file 4: Figure S1.** Assessment of the specificity of the triplex *cox*1-based qPCR in detecting the target DNA.

**Additional file 5: Table S4.** Analytical sensitivity of the pan-filarial *28S*-based qPCR in detecting single-species DNA.

**Additional file 6: Figure S2. a**, **b**, **c**-1. Efficiency of pan-filarial *28S*-based qPCR using single-species DNA of *D. immitis, D. repens* and *A. reconditum*. **a**, **b**, **c**-2. Standard curves generated from serial 10-fold dilution of each DNA.

**Additional file 7: Table S5.** Analytical sensitivity of the triplex *cox*1-based qPCR using pooled DNA.

**Additional file 8: Figure S3. a** Efficiency of the triplex *cox*1-based qPCR using pooled DNA. **b** Standard curves generated from serial 10-fold dilution of DNA.

**Additional file 9: Table S6.** Analytical sensitivity of the triplex *cox*1 and the duplex *ftsZ*-based qPCRs in detecting single-species DNA of *D. immitis* and its *Wolbachia*.

**Additional file 10: Figure S4. a, b** Efficiency of the triplex *cox*1 and the duplex *ftsZ*-based qPCRs using single-species DNA of *D. immitis* and its *Wolbachia*. **c**, **d** Standard curves generated from serial 10-fold dilution of DNA.

**Additional file 11: Table S7.** Samples distribution according to filariae and *Wolbachia* DNA detected by the sequence typing approach.

**Additional file 12: Table S8.** Samples distribution according to filariae and *Wolbachia* DNA detected by the multiplex approach.

**Additional file 13: Table S9.** Samples tested positive by at least by one diagnostic approach.

**Additional file 14: Table S10.** Performance of molecular and serological assays in detecting *D.* *immitis* infection.

**Additional file 15: Table S11.** Performance of molecular approaches in detecting *D. repens* and *A. reconditum*.

## Data Availability

The data supporting the conclusions of this article are included within the article and its additional files.

## Abbreviations

FAM: 6-FAM (6-carboxyfluorescein)
VIC: VIC (2′-chloro-phenyl-1,4- dichloro-6-carboxyfluorescein)
Cy5: cyanine 5
TAMRA: tetra-methyl-rhodamine
BHQ: black hole quencher
