# Supplementary material for: Development of a multiplex qPCR-based approach for the diagnosis of Dirofilaria immitis, D. repens and Acanthocheilonema reconditum
Source: Parasit Vectors. 2020 Jun 22;13:319. doi: 10.1186/s13071-020-04185-0 (PMC7309989; doi:10.1186/s13071-020-04185-0)
Supplement: Supplementary file 1 — Additional file 1: Table S1. DNA used as a positive control in this study. [file 13071_2020_4185_MOESM1_ESM.docx]

**Additional file 1: Table S1.** DNA used as positive control in this study.

| N° | **Species name** | **Isolation source** | **Host** | **Country** |
| --- | --- | --- | --- | --- |
| 1 | *Dirofilaria immitis* | Adult worm from pulmonary arteries | Dog | Indre, France |
| 2 | *Dirofilaria repens* | Adult worm in subcutaneous nodules | Dog | Pau, France |
| 3 | *Dirofilaria repens* | Adult worm (peritoneal cavity) | Donkey | Egypt |
| 4 | *Acanthocheilonema reconditum* | Blood | Dog | Côte d'Ivoire |
| 5 | *Onchocerca lupi* | Skin | Dog | Courtesy of Prof D. Otranto, Bari, Italy |
| 6 | *Thelazia callipaeda* | Eye | Dog | Courtesy of Prof D. Otranto, Bari, Italy |
| 7 | *Cercopithifilaria bainae* | Blood | Dog | French Guiana |
| 8 | *Acanthocheilonema* sp. | Blood | Dog | Egypt |
| 9 | *Dipetalonema* sp. | Blood | Horse | Egypt |
| 10 | *Dipetalonema* sp. | Adult worm (peritoneal cavity) | Donkey | Egypt |
| 11 | *Brugia malayi* | Infected *Aedes aegypti* | Laboratory strain | University of Georgia College of Veterinary Medicine, Athens, USA |
| 12 | *Brugia pahangi* | Infected *Ae. aegypti* | Laboratory strain | University of Georgia College of Veterinary Medicine, Athens, USA |
| 13 | *Brugia* sp. | Blood | Howler monkeys | French Guiana |
| 14 | *Wuchereria bancrofti* | Blood | Human | Côte d'Ivoire |
| 15 | *Loa loa* | Blood | Gabonese children | Gabon |
| 16 | *Mansonella perstens* | Blood | Febrile patient | Senegal |
| 17 | *Onchocerca* sp. | Blood | Howler monkeys | French Guiana |
| 18 | *Setaria digitata* | Adult worm (peritoneal cavity) | Donkey | Egypt |
| 19 | *Dipetalonema* sp. | Blood | Donkey | Egypt |
| 20 | *Wolbachia of Ae. albopictus* | *Adult mosquitoes* | Laboratory *Ae. albopictus* | Marseille, France |
| 21 | *Wolbachia of An. gambiae* | Cell culture | Laboratory *An. gambiae* | Marseille, France |
| 22 | *Wolbachia of Cimex lectularius* | Cell culture | Laboratory *C. lectularius* | Marseille, France |
| 23 | *Wolbachia of C. hemipterus* | Cell culture | Laboratory *C. hemipterus* | Marseille, France |
| 24 | *Wolbachia of D. immitis* | Dog blood microfilariae | *D. immitis* | Corsica Island, France |
| 25 | *Wolbachia of D. repens* | Dog blood microfilariae | *D. repens* | France |
| 26 | *Wolbachia of O. lupi* | Dog skin microfilariae | *O. lupi* | Courtesy of Prof D. Otranto, Bari, Italy |
| 27 | *Wolbachia of W. bancrofti* | Human blood microfilariae | *W. bancrofti* | Côte d'Ivoire |
| 28 | *Wolbachia of Brugia* sp. | Monkey blood microfilariae | *Brugia* sp. | French Guiana |
