# Supplementary material for: Development of a multiplex qPCR-based approach for the diagnosis of Dirofilaria immitis, D. repens and Acanthocheilonema reconditum
Source: Parasit Vectors. 2020 Jun 22;13:319. doi: 10.1186/s13071-020-04185-0 (PMC7309989; doi:10.1186/s13071-020-04185-0)
Supplement: Supplementary file 3 — Additional file 3: Table S3.In vitro validation of the triplex cox1-based qPCR. [file 13071_2020_4185_MOESM3_ESM.docx]

**Table S3:** The specificity of the triplex *cox*1-based qPCR was challenged in vitro against the single-species and pooled DNA of *D. immitis*, *D. repens* and *A. reconditum*.

**Additional file 3: Table S3.** *In vitro* validation of the triplex *cox*1-based qPCR.

| **Single/pooled DNAs** | **Relative fluorescence units recorded at the end of qPCR run** | | | **Positive signal** |
| --- | --- | --- | --- | --- |
|  | **FAM** | **VIC** | **Cy5** |  |
| *D.immitis* | 2619 | 49.6 | 5.13 | FAM |
| *D.repens* | 71 | 3085 | 9.23 | VIC |
| *A. reconditum* | 19.5 | 56.1 | 1583 | Cy5 |
| *D.immitis, D.repens* and *A. reconditum* | 3603 | 2973 | 375 | FAM-VIC-Cy5 |
| Cut Off Value | 351.3 | 357.3 | 99.2 | // |
| Negative control | 35.7 | 49.1 | 0.589 | // |

**FAM**: Absorption channels for 6-FAM (6-Carboxyfluorescein), **VIC**: Absorption channels for VIC (2'-chloro-phenyl-1,4- dichloro-6-carboxyfluorescein), **Cy5**: Absorption channels for Cyanine 5.
