# Supplementary figures and images for: Development of a multiplex qPCR-based approach for the diagnosis of Dirofilaria immitis, D. repens and Acanthocheilonema reconditum
Source: Parasit Vectors. 2020 Jun 22;13:319. doi: 10.1186/s13071-020-04185-0 (PMC7309989; doi:10.1186/s13071-020-04185-0)

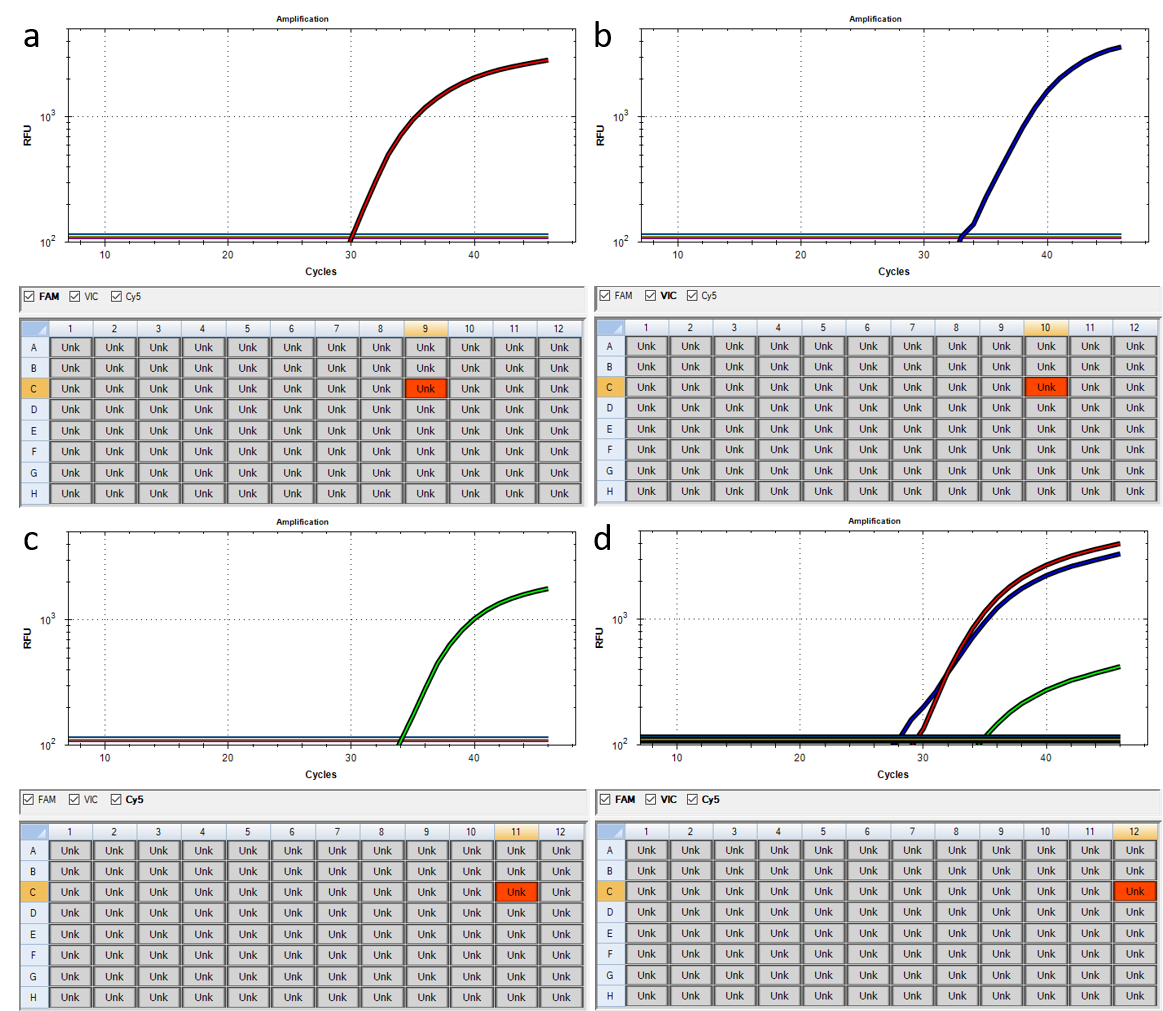

Supplement: Supplementary file 4 — Additional file 4: Figure S1. Assessment of the specificity of the triplex cox1-based qPCR in detecting the target DNA. [file 13071_2020_4185_MOESM4_ESM.tif]

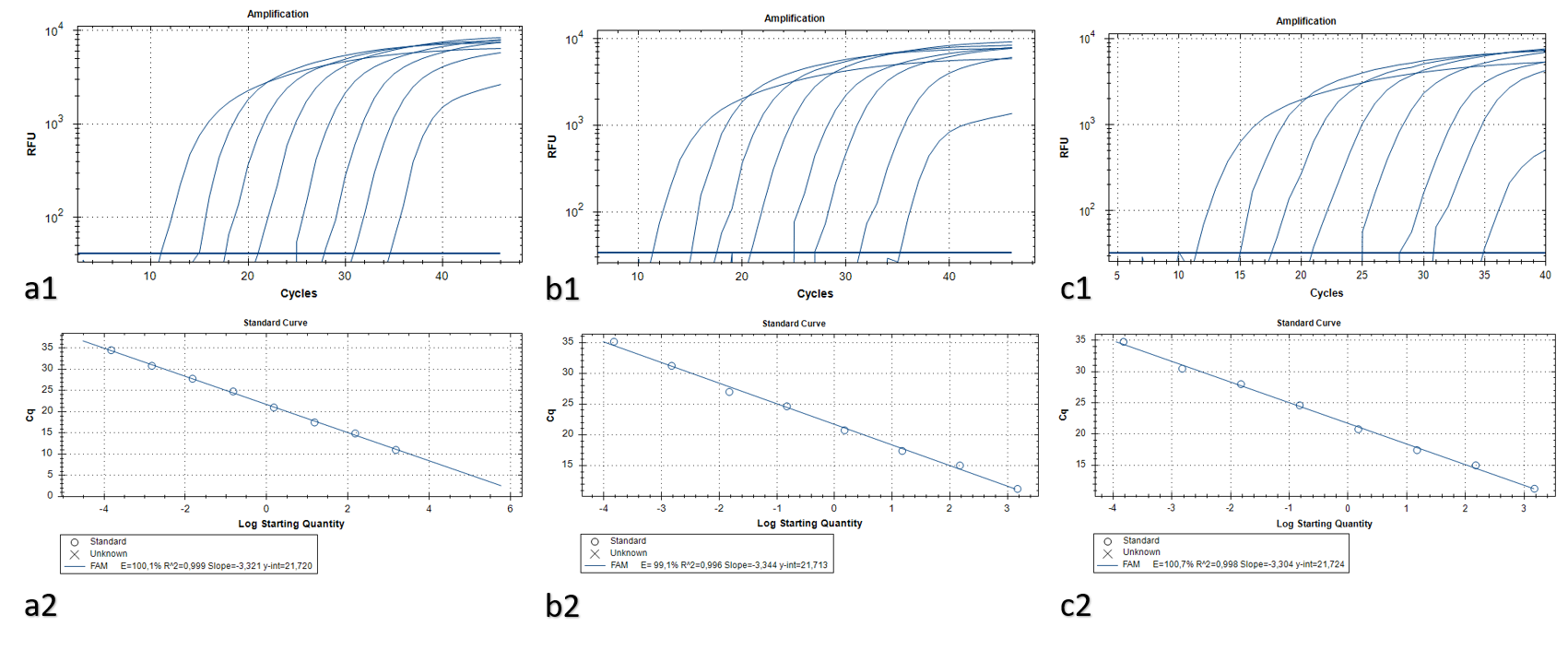

Supplement: Supplementary file 6 — Additional file 6: Figure S2. a, b, c-1. Efficiency of pan-filarial 28S-based qPCR using single-species DNA of D. immitis, D. repens and A. reconditum. a, b, c-2. Standard curves generated from serial 10-fold dilution of each DNA. [file 13071_2020_4185_MOESM6_ESM.tif]

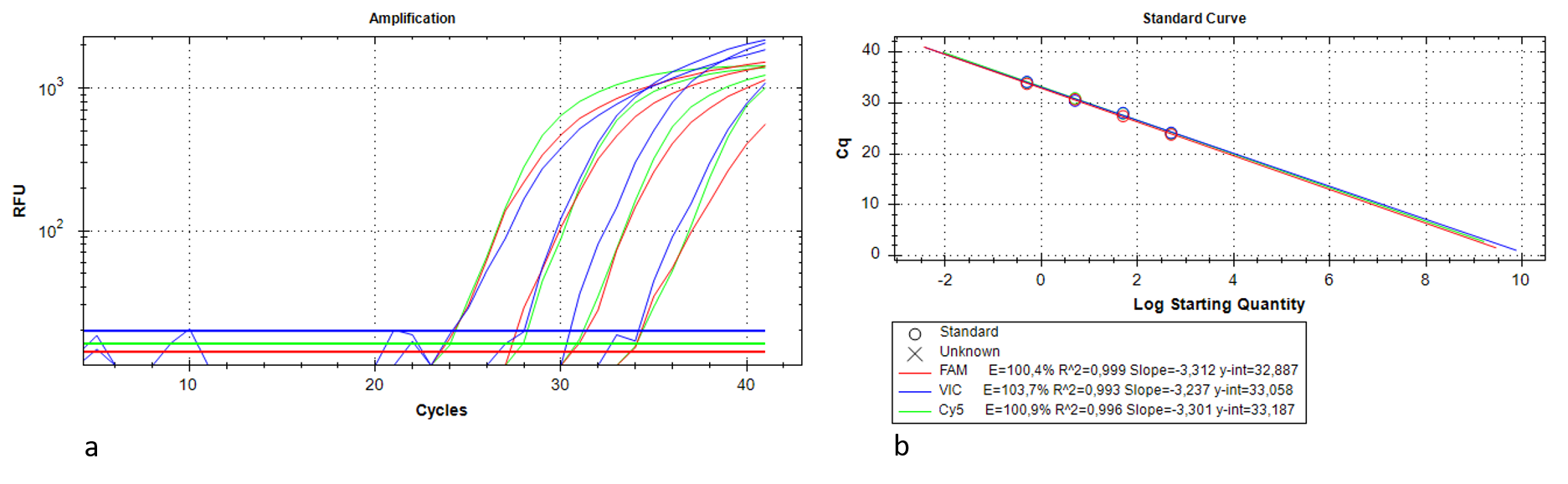

Supplement: Supplementary file 8 — Additional file 8: Figure S3. a Efficiency of the triplex cox1-based qPCR using pooled DNA. b Standard curves generated from serial 10-fold dilution of DNA. [file 13071_2020_4185_MOESM8_ESM.tif]

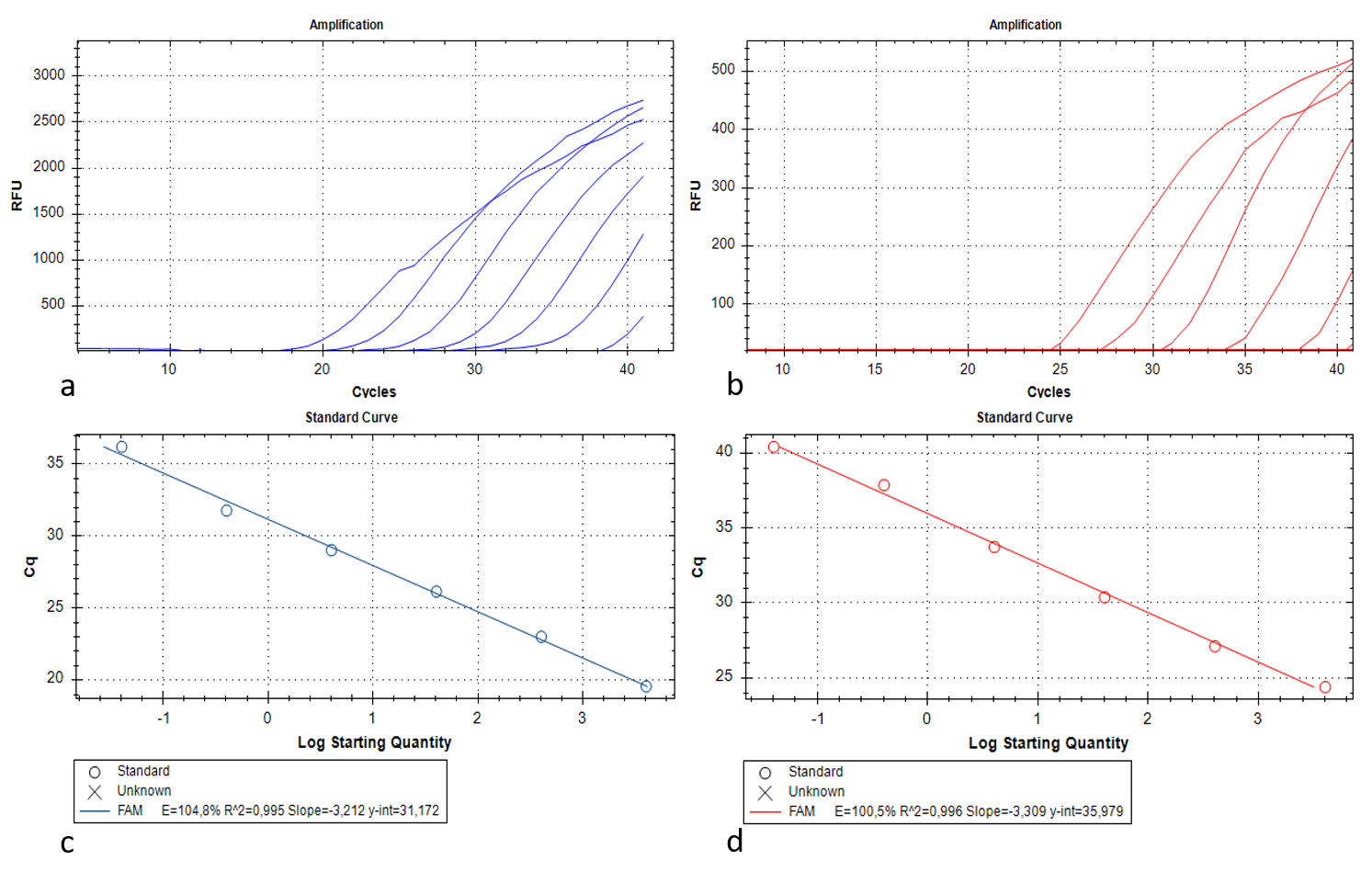

Supplement: Supplementary file 10 — Additional file 10: Figure S4. a, b Efficiency of the triplex cox1 and the duplex ftsZ-based qPCRs using single-species DNA of D. immitis and its Wolbachia. c, d Standard curves generated from serial 10-fold dilution of DNA. [file 13071_2020_4185_MOESM10_ESM.tif]
