# Supplementary material for: Development of a multiplex qPCR-based approach for the diagnosis of Dirofilaria immitis, D. repens and Acanthocheilonema reconditum
Source: Parasit Vectors. 2020 Jun 22;13:319. doi: 10.1186/s13071-020-04185-0 (PMC7309989; doi:10.1186/s13071-020-04185-0)
Supplement: Supplementary file 5 — Additional file 5: Table S4. Analytical sensitivity of the pan-filarial 28S-based qPCR in detecting single-species DNA. [file 13071_2020_4185_MOESM5_ESM.docx]

**Table S4:** Analytical sensitivity and detection limit of the pan-filarial 28S-based qPCR assay were assessed by using a serial 10-fold dilution of a single-species DNA of D. immitis, D. repens and A. reconditum. PCR efficiency, slope, Y-intercept and correlation coefficient were generated to evaluate the qPCR reaction.

**Additional file 5: Table S4. .** Analytical sensitivity of the pan-filarial *28S*-based qPCR in detecting single-species DNA.

| **Microfilaria load** | | ***D. immitis DNA*** | | | ***D. repens DNA*** | | | ***A. reconditum DNA*** | | |
| --- | --- | --- | --- | --- | --- | --- | --- | --- | --- | --- |
| **Per qPCR reaction (mf/5µl of DNA)** | **Per ml of blood** | **Ct** | **E-RFU** | **SCRS** | **Ct** | **E-RFU** | **SCRS** | **Ct** | **E-RFU** | **SCRS** |
| 0.75 × 10^+1^ | 1.5 × 10^+3^ | 12.32 | 6127 | (E= 100.1%)  (S=-3.32)  (Y.int=21.72)  (R2=0.999) | 11.26 | 5938 | (E=99.1%)  (S=-3.34)  (Y.int=21.71)  (R2=0.996) | 11.26 | 5667 | (E=100.7%)  (S=-3.304)  (Y.int=21.72)  (R2=0.998) |
| 0.75 × 10^+0^ | 1.5 × 10^+2^ | 16.1 | 7420 |  | 15 | 7802 |  | 15 | 7619 |  |
| 0.75 × 10^-1^ | 1.5 × 10^+1^ | 19.44 | 7690 |  | 17.44 | 8399 |  | 17.44 | 7825 |  |
| 0.75 × 10^-2^ | 1.5 × 10^+0^ | 22.82 | 8221 |  | 20.78 | 9037 |  | 20.78 | 8270 |  |
| 0.75 × 10^-3^ | 1.5 × 10^-1^ | 26.12 | 7660 |  | 24.6 | 7623 |  | 24.6 | 7794 |  |
| 0.75 × 10-^4^ | 1.5 × 10^-2^ | 29.58 | 7024 |  | 28 | 7389 |  | 28 | 6402 |  |
| 0.75 × 10^-5^ | 1.5 × 10^-3^ | 32.23 | 5360 |  | 30.48 | 5595 |  | 30.48 | 5435 |  |
| 0.75 × 10^-6^ | 1.5 × 10^-4^ | 34.66 | 2325 |  | 34.78 | 1226 |  | 34.78 | 2625 |  |
| Cut Off Value | // | 35 | 909 |  | 35 | 909 |  | 35 | 909 |  |
| Negative Control | // | N/A | 10.1 |  | N/A | 10.1 |  | N/A | 10.1 |  |

**mf**: microfilariae, **Ct:** Cycle threshold, **N/A**: No amplification, **E-RFU**: End of relative fluorescence unit, **SCRS**: Standard Curve Results Spreadsheet, **E**: Efficiency, **S**: Slope, **Y.int:** Y-intercept.
