# Supplementary material for: Development of a multiplex qPCR-based approach for the diagnosis of Dirofilaria immitis, D. repens and Acanthocheilonema reconditum
Source: Parasit Vectors. 2020 Jun 22;13:319. doi: 10.1186/s13071-020-04185-0 (PMC7309989; doi:10.1186/s13071-020-04185-0)
Supplement: Supplementary file 7 — Additional file 7: Table S5. Analytical sensitivity of the triplex cox1-based qPCR using pooled DNA. [file 13071_2020_4185_MOESM7_ESM.docx]

**Table S5.** Analytical sensitivity and detection limit of the triplex *cox*1-based qPCR were assessed using a serial 10-fold dilution of pooled DNA of pooled DNA of *D. immitis, D. repens* and *A. reconditum.* PCR efficiency, slope, Y-intercept and correlation coefficient were generated to evaluate the qPCR reaction.

**Additional file 7: Table S5.** Analytical sensitivity of the triplex *cox*1-based qPCR using pooled DNA.

| **Microfilaria load** | | ***D. immitis* DNA** | | | ***D. repens* DNA** | | | ***A. reconditum* DNA** | | |
| --- | --- | --- | --- | --- | --- | --- | --- | --- | --- | --- |
| **Per qPCR reaction (mf/5µl of DNA)** | **Per milliliter of blood** | **Ct** | **FAM** | **SCRS** | **Ct** | **VIC** | **SCRS** | **Ct** | **Cy-5** | **SCRS** |
| 2.5 × 10^+0^ | 5.00 × 10^+2^ | 23.81 | 1387 | (E= 100.4%)  (S=-3.312)  (Y.int= 32.89)  (R^2^=0.999) | 24.13 | 1590 | (E=103.7%)  (S=-3.237)  (Y.int=33.058)  (R^2^=0.993) | 24.03 | 1405 | E=100.9%)  (S=-3.30)  (Y.int=33.19)  (R^2^=0.996) |
| 2.5 × 10^-1^ | 5.00 × 10^+1^ | 27.41 | 1241 |  | 28.01 | 1852 |  | 27.94 | 1302 |  |
| 2.5 × 10^-2^ | 5.00 × 10^+0^ | 30.7 | 865 |  | 30.45 | 1614 |  | 30.9 | 1009 |  |
| 2.5 × 10^-3^ | 5.00 × 10^-1^ | 33.75 | 297 |  | 34.11 | 567 |  | 34.05 | 512 |  |
| 2.5 × 10^-4^ | 5.00 × 10^-2^ | 35.83 | 193 |  | 36.45 | 66.1 |  | 38.74 | 11.3 |  |
| Cut Off Value | // | 35 | 200 |  | 35 | 73.9 |  | 35 | 40.2 |  |
| Negative Control | // | // | 10.5 |  | // | 15 |  | // | 1.5 |  |

**mf:** Microfilaria, **Ct:** Cycle threshold, **SCRS**: Standard Curve Results Spreadsheet, **E**: Efficiency, **S**: Slope, **Y.int:** Y-intercept.
