# Supplementary material for: Development of a multiplex qPCR-based approach for the diagnosis of Dirofilaria immitis, D. repens and Acanthocheilonema reconditum
Source: Parasit Vectors. 2020 Jun 22;13:319. doi: 10.1186/s13071-020-04185-0 (PMC7309989; doi:10.1186/s13071-020-04185-0)
Supplement: Supplementary file 9 — Additional file 9: Table S6. Analytical sensitivity of the triplex cox1 and the duplex ftsZ-based qPCRs in detecting single-species DNA of D. immitis and its Wolbachia. [file 13071_2020_4185_MOESM9_ESM.docx]

**Table S6.** Analytical sensitivity and detection limit of the triplex *cox*1-based qPCR were assessed using a serial 10-fold dilution of a single-species DNA of *D. immitis* and its *Wolbachia.* PCR efficiency, slope, Y-intercept and correlation coefficient were generated to evaluate the qPCR reaction.

**Additional file 9: Table S6.** Analytical sensitivity of the triplex *cox*1 and the duplex *ftsZ*-based qPCRs in detecting single-species DNA of *D. immitis* and its *Wolbachia*.

| **Microfilaria load** | | ***D. immitis* DNA** | | | ***Wolbachia* of *D. immitis* DNA** | | |
| --- | --- | --- | --- | --- | --- | --- | --- |
| **Per qPCR reaction (mf/5µl of DNA)** | **Per milliliter of blood** | **Ct** | **E-RFU** | **SCRS** | **Ct** | **E-RFU** | **SCRS** |
| 2.01 × 10^+2^ | 4,03 × 10^+3^ | 18.11 | 2383 | (E=104.8%)  (S=-3.212)  (Y.int= 31.172)  (R^2=0.995) | 24.39 | 497 | (E=100.5%)  (S=-3.309)  (Y.int=35.98)  (R^2=0.996) |
| 2.01 × 10^+1^ | 4,03 × 10^+2^ | 21.03 | 2591 |  | 27.11 | 450 |  |
| 2.01 × 10^+0^ | 4,03 × 10^+1^ | 24.66 | 2448 |  | 30.38 | 455 |  |
| 2.01 × 10^-1^ | 4,03 × 10^+0^ | 27.54 | 2006 |  | 33.74 | 272 |  |
| 2.01 × 10^-2^ | 4,03 × 10^-1^ | 29.23 | 1504 |  | 37.87 | 67 |  |
| 2.01 × 10^-3^ | 4,03 × 10^-2^ | 30.93 | 773 |  | 40.42 | 14.9 |  |
| 2.01 × 10^-4^ | 4,03 × 10^-3^ | 35.28 | 125 |  | N/A | N/A |  |
| 2.01 × 10^-5^ | 4,03 × 10^-4^ | 38.39 | 21 |  | N/A | N/A |  |
| Cut Off value | // | 35 | 158 |  | 38 | 30.5 |  |
| Negative Control | // | // | 8.68 |  | // | 5 |  |

**mf:** Microfilaria, **Ct:** Cycle threshold, **N/A**: No amplification, **RFU**: relative fluorescence unit, **SCRS**: Standard Curve Results Spreadsheet, **E**: Efficiency, **S**: Slope, **Y.int:** Y-intercept.
