# Supplementary material for: Development of a multiplex qPCR-based approach for the diagnosis of Dirofilaria immitis, D. repens and Acanthocheilonema reconditum
Source: Parasit Vectors. 2020 Jun 22;13:319. doi: 10.1186/s13071-020-04185-0 (PMC7309989; doi:10.1186/s13071-020-04185-0)
Supplement: Supplementary file 11 — Additional file 11: Table S7. Samples distribution according to filariae and Wolbachia DNA detected by the sequence typing approach. [file 13071_2020_4185_MOESM11_ESM.docx]

**Additional file 11: Table S7.** Samples distribution according to filariae and *Wolbachia* DNA detected by the sequence typing approach.

| **Molecular markers detected per sample or group of samples** | **Number of positive samples** | **Sample names** |
| --- | --- | --- |
| *A. reconditum*  *D. repens*  *Wolbachia* of *D. immitis* | 1 | mf-CI-122 |
| *D. immitis*  Untyped *Wolbachia* | 1 | CI-EA-64 |
| *D. immitis*  *Wolbachia* of *D. immitis* | 9 | CI-EA-136 mf-MWD19 CI-EA-26 mf-MWD03 CI-EA-135 CI-EA-01 CI-EA-03 CI-EA-31 CI-EA-132 |
| *D. repens*  Untyped *Wolbachia* | 3 | mf-MWD05 mf-MWD02 mf-MWD09 |
| *D. repens*  *Wolbachia* of *D. immitis* | 1 | CI-EA-120 |
| *D. repens*  *Wolbachia* of *D. repens* | 1 | CI-EA-56 |
| *A. reconditum*  *Wolbachia* of *D. repens* | 1 | mf-CI-97 |
| Untyped *Wolbachia*  Untyped filaria | 1 | mf-MWD01 |
| Untyped filaria  *Wolbachia* of *D. immitis* | 3 | CI-EA-122 CI-EA-91 CI-EA-55 |
| *D. immitis* | 2 | CI-EA-97 CI-EA-27 |
| *D. repens* | 2 | CI-EA-62 CI-EA-124 |
| *A. reconditum* | 14 | mf-CI-50 mf-CI-109 mf-CI-28 mf-CI-118 mf-CI-101 mf-CI-110 mf-CI-115 mf-CI-104 mf-CI-111 mf-CI-116 mf-CI-102 mf-CI-98 mf-CI-99 mf-CI-96 |
| Untyped filaria | 1 | mf-CI-123 |
| *Wolbachia* of *D. immitis* | 8 | CI-EA-121 CI-EA-111 CI-EA-119 CI-EA-116 CI-EA-113 CI-EA-94 CI-EA-117 CI-EA-114 |
| *Wolbachia* of *D. repens* | 1 | Ms-MWD09 |
