# Supplementary material for: Development of a multiplex qPCR-based approach for the diagnosis of Dirofilaria immitis, D. repens and Acanthocheilonema reconditum
Source: Parasit Vectors. 2020 Jun 22;13:319. doi: 10.1186/s13071-020-04185-0 (PMC7309989; doi:10.1186/s13071-020-04185-0)
Supplement: Supplementary file 14 — Additional file 14: Table S10. Performance of molecular and serological assays in detecting D. immitis infection. [file 13071_2020_4185_MOESM14_ESM.docx]

**Additional file 14: Table S10.** Performance of molecular and serological assays in detecting *D. immitis* infection.

| **Assay characteristics** | **Molecular approaches** | | **Heartworm antigen test** | |
| --- | --- | --- | --- | --- |
|  | **Sequence typing** | **Multiplex** | **B h-Tx** | **A h-Tx** |
| Correct classification | 96.4 ± 5.6 | 99.4 ± 0.9 | 88.7 ± 4.8 | 45.8 ± 7.5 |
| Misclassification | 3.6 ± 5.6 | 0.6 ± 0.9 | 11.3 ± 4.8 | 54.2 ± 7.5 |
| Sensitivity | 82.8 ± 29.9 | 100.0 ± 6.0 | 65.5 ± 18.2 | 100.0 ± 6.0 |
| Specificity | 99.3 ± 3.9 | 99.3 ± 2.0 | 93.5 ± 4.5 | 34.5 ± 8.2 |
| False positive rate | 0.7 ± 2.1 | 0.7 ± 1.1 | 6.5 ± 4.1 | 65.5 ± 7.9 |
| False negative rate | 17.2 ± 27.5 | 0.0 ± 0.0 | 34.5 ± 17.3 | 0.0 ± 0.0 |
| True prevalence | 17.3 ± 11.4 | 17.3 ± 5.7 | 17.3 ± 5.7 | 17.3 ± 5.7 |
| Apparent prevalence | 14.88 | 17.85 ± | 16.67 | 71.43 |
| PPV (Positive Predictive Value) | 96.0 ± 11.7 | 96.7 ± 4.9 | 67.9 ± 17.3 | 24.2 ± 7.7 |
| NPV (Negative Predictive Value) | 96.5 ± 6.0 | 100 ± 0.0 | 92.9 ± 4.3 | 100.0 ± 0.0 |
| Cohen's Kappa | 0.87 | 0.98 | 0.60 | 0.15 |
| Agreement | almost perfect | almost perfect | moderate | slight |

**B h-Tx**: Before heat pre-treatment of sera; **A h-Tx**: After heat pre-treatment of sera*.*
