# Supplementary material for: Development of a multiplex qPCR-based approach for the diagnosis of Dirofilaria immitis, D. repens and Acanthocheilonema reconditum
Source: Parasit Vectors. 2020 Jun 22;13:319. doi: 10.1186/s13071-020-04185-0 (PMC7309989; doi:10.1186/s13071-020-04185-0)
Supplement: Supplementary file 15 — Additional file 15: Table S11. Performance of molecular approaches in detecting D. repens and A. reconditum. [file 13071_2020_4185_MOESM15_ESM.docx]

**Additional file 15: Table S11.** Performance of molecular approaches in detecting *D. repens* and *A. reconditum*.

| **Assay characteristics** | ***D. repens*** | | ***A. reconditum*** | |
| --- | --- | --- | --- | --- |
|  | **Sequence typing approach** | **Multiplex approach** | **Sequence typing approach** | **Multiplex approach** |
| Correct classification | 96.4 ± 5.6 | 100.0 ± 0.0 | 99.4 ± 0.9 | 100.0 ± 0.0 |
| Misclassification | 3.6 ± 5.6 | 0.0 ± 0.0 | 0.6 ± 0.9 | 0.0 ± 0.0 |
| Sensitivity | 62.5 ± 49.4 | 100.0 ± 10.3 | 94.1 ± 14.3 | 100.0 ± 9.8 |
| Specificity | 100.0 ± 2.4 | 100.0 ± 1.2 | 100.0 ± 1.2 | 100.0 ± 1.2 |
| False positive rate | 0.0 ± 0.0 | 0.0 ± 0.0 | 0.0 ± 0.0 | 0.0 ± 0.0 |
| False negative rate | 37.5 ± 47.4 | 0.0 ± 0.0 | 5.9 ± 8.5 | 0.0 ± 0.0 |
| Prevalence | 9.5 ± 8.9 | 9.5 ± 4.4 | 10.1 ± 4.6 | 10.1 ± 4.6 |
| PPV (Positive Predictive Value) | 100.0 ± 0.0 | 100.0 ± 0.0 | 100.0 ± 0.0 | 100.0 ± 0.0 |
| NPV (Negative Predictive Value) | 96.2 ± 6.0 | 100.0 ± 0.0 | 99.3 ± 1.0 | 100.0 ± 0.0 |
| Cohen's Kappa | 0.75 | 1.00 | 0.97 | 1.00 |
| Agreement | substantial | almost perfect | almost perfect | almost perfect |
